# Alterations in iron content, iron-regulatory proteins and behaviour without tau pathology at one year following repetitive mild traumatic brain injury

**DOI:** 10.1186/s40478-023-01603-z

**Published:** 2023-07-18

**Authors:** Sydney M. A. Juan, Maria Daglas, Phan H. Truong, Celeste Mawal, Paul A. Adlard

**Affiliations:** Synaptic Neurobiology Laboratory, The Florey Institute of Neuroscience and Mental Health, The Melbourne Dementia Research Centre, The University of Melbourne, Kenneth Myer Building, 30 Royal Parade, Parkville, Melbourne, VIC 3052 Australia

**Keywords:** Repetitive mild traumatic brain injury, Tau phosphorylation, Metal dyshomeostasis, Iron-regulatory proteins, Neurodegeneration

## Abstract

Repetitive mild traumatic brain injury (r-mTBI) has increasingly become recognised as a risk factor for the development of neurodegenerative diseases, many of which are characterised by tau pathology, metal dyshomeostasis and behavioural impairments. We aimed to characterise the status of tau and the involvement of iron dyshomeostasis in repetitive controlled cortical impact injury (5 impacts, 48 h apart) in 3-month-old C57Bl6 mice at the chronic (12-month) time point. We performed a battery of behavioural tests, characterised the status of neurodegeneration-associated proteins (tau and tau-regulatory proteins, amyloid precursor protein and iron-regulatory proteins) via western blot; and metal levels using bulk inductively coupled plasma-mass spectrometry (ICP-MS). We report significant changes in various ipsilateral iron-regulatory proteins following five but not a single injury, and significant increases in contralateral iron, zinc and copper levels following five impacts. There was no evidence of tau pathology or changes in tau-regulatory proteins following five impacts, although some changes were observed following a single injury. Five impacts resulted in significant gait deficits, mild anhedonia and mild cognitive deficits at 9–12 months post-injury, effects not seen following a single injury. To the best of our knowledge, we are the first to describe chronic changes in metals and iron-regulatory proteins in a mouse model of r-mTBI, providing a strong indication towards an overall increase in brain iron levels (and other metals) in the chronic phase following r-mTBI. These results bring to question the relevance of tau and highlight the involvement of iron dysregulation in the development and/or progression of neurodegeneration following injury, which may lead to new therapeutic approaches in the future.

**Supplementary Information:**

The online version contains supplementary material available at 10.1186/s40478-023-01603-z.

## Introduction

Traumatic brain injury (TBI) results from an external force that contacts the head and leads to brain damage, commonly occurring during vehicle accidents, sports injuries, blast wave trauma, assaults and falls [1, 2]. It is estimated that 54–60 million individuals experience a TBI each year globally [3]. TBI results in a complex cascade of interconnected biochemical events such as neuronal death [4], axonal injury [5], protein aggregation [6], metal dyshomeostasis [7, 8], excitotoxicity [9] and calcium overload [10], often leading to long-lasting cognitive, personality and functional impairments [11]. Unfortunately, there are currently no approved treatments that effectively target the pathophysiological mechanisms following TBI.

In recent years, the incidence of repetitive mild traumatic brain injury (r-mTBI) particularly within sports settings has gained significant scientific, media and public attention. Importantly, r-mTBI is increasingly being recognised as a risk factor for the development of several neurodegenerative diseases such as Alzheimer’s disease (AD) [12], Parkinson’s disease (PD) [13] and more recently Chronic Traumatic Encephalopathy (CTE) [14]. CTE is defined as a chronic and progressive neurodegenerative condition characterised by cognitive, motor and personality changes and tau pathology [15]. Tau is a microtubule stabilizing protein [16] that is normally phosphorylated, but under pathological conditions (such as in AD, CTE and following TBI), tau can become hyperphosphorylated eventually leading to neuronal death and cognitive impairment [17]. The phosphorylation state of tau is tightly controlled by kinase proteins such as glycogen synthase kinase (GSK3β), cycline-dependent kinase 5 (CDK5), extracellular signal-regulated kinases (ERK1/2) and protein kinase C (PKC) which function to phosphorylate tau, and phosphatase proteins such as protein phosphatase 1 (PP1), protein phosphatase 2 A (PP2A) and protein phosphatase 2B (PP2B) [18] which function to dephosphorylate tau. As such, aberrant tau pathology is associated with a dysregulation of the kinase/phosphatase system [19]. The acute period following injury has been extensively characterised in the literature both in clinical [20–22] and pre-clinical settings using a wide range of animal models and experimental conditions [23–25]. Yet to determine whether animal models of r-mTBI actually lead to the behavioural and pathological hallmarks true to the human condition, a characterisation of the chronic outcomes following r-mTBI is needed. One of the major problems facing the field is that differences in experimental design and injury parameters have led to varying results and hence a lack of consensus regarding the involvement of tau in r-mTBI. Indeed, while some animal studies report the presence of tau pathology long after the last impact [26, 27], others report a lack of tau phosphorylation at chronic time points post-injury [28, 29]. As such, the inconsistency in the results brings to question the involvement of tau in driving disease pathology and consequently whether better animal models of r-mTBI are needed.

Furthermore, there has been a surprising lack of focus on the potential involvement of metal ion dyshomeostasis (such as iron, zinc and copper) in the development and/or progression of neurodegeneration following r-mTBI. Indeed, metal ion dyshomeostasis has been widely studied in the context of neurodegenerative diseases and is a prominent feature of ageing [30–32] and in the development of AD [33] and PD [34]. The levels and distribution of metals in the brain are normally controlled by metal-regulatory proteins. For instance, iron is endocytosed by transferrin receptor (TfR), which mediates the endocytosis of transferrin (Tf) bound iron into the cell [35]. Once in the endosome, iron is transported into the cytosol by divalent metal transporter-1 (DMT1), which transports iron across cellular membranes [36], where it can either be stored as ferritin, the major iron storage protein [37], or exported out of the cell by ferroportin [38]. These iron-regulatory proteins therefore respond to changes in overall iron content in the brain. Metals have been shown to play a role in protein folding [39] and studies have shown that they can directly bind to tau [40]. We previously showed that an rTg4510 mouse model of tauopathy treated with the copper/zinc chaperone drug PBT2 had a reduction in tau aggregation, concomitant with an increase in phosphatase proteins, and improvement in cognition [41]. We have also previously shown that various metals (particularly iron) are altered following a single TBI [42], but whether this extends to repetitive injuries has yet to be studied, highlighting an important gap in the literature given the propensity for r-mTBI to lead to neurodegenerative disease. Lastly, using a well-established mouse model of r-mTBI [43] in young-adult (4-month-old) mice, we have recently demonstrated that brain iron levels are significantly elevated in the sub-acute (1-month) period following r-mTBI [8]. Additionally, these mice displayed behavioural deficits and changes in proteins involved in neurodegeneration pathways such as the amyloid precursor protein (APP) and various tau-regulatory proteins [44]. Therefore, the potential involvement of iron and its regulatory proteins in the development and/or progression of neurodegeneration, which characterises the chronic outcomes of r-mTBI, warrants further investigation.

Therefore, the aim of this study was to investigate whether a single or five repeated injuries would result in (1) transcriptional alterations in molecular pathways associated with neurodegeneration, (2) behavioural deficits characteristic of human r-mTBI, (3) changes in metal levels (with a particular focus on iron) and (4) translational changes via altered expression of proteins involved in neurodegeneration (such as APP, tau-regulatory proteins, various tau proteins/species and iron-regulatory proteins) at a chronic (12-month) time point post-injury. We performed bulk RNA sequencing following r-mTBI at the chronic 6-month time point post-injury. We performed a battery of behavioural tests to examine neurological, locomotor, gait, spatial learning and memory and short-term memory function, as well as anxiety and depressive-like behaviour at various time points post-injury. Metal analysis was conducted using inductively coupled plasma-mass spectrometry (ICP-MS) and protein analysis via western blot at 12-months post-injury. We hypothesised that five repeated impacts would result in the differential expression of neurodegeneration-associated molecular pathways at 6-months post-injury, and significant behavioural deficits, an increase in tau phosphorylation and total iron content as well as alterations in tau and iron-regulatory proteins at 12-months post-injury.

## Materials and methods

### Animals

C57Bl6 male and female mice at 12 weeks of age were purchased from the Animal Resources Centre (ARC, Western Australia). Animals were group housed (5 mice per cage) in Techniplast IVC cages under standard laboratory conditions (12-hour light/dark cycle) and were given access to food and water *ad libitum.* The mice were acclimatised to the holding room and handled by the experimenter for a minimum of one week prior to the start of any experimental procedure. All animal experimental procedures were approved by the Florey Institute of Neuroscience and Mental Health (FINMH) Animal Ethics Committee and were conducted in accordance with the Prevention of Cruelty to Animals Act and Code of Practice for the Use of Animals for Scientific Purposes as described by the National Health and Medical Research Council of Australia.

### Experimental design

**Fig. 1:**
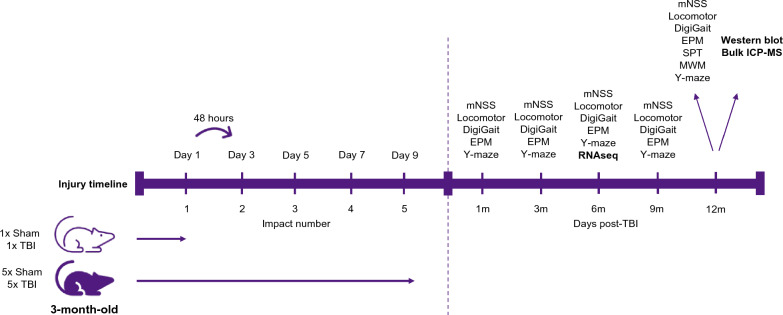
Experimental timeline and design. Timeline of injury induction for the experimental groups: 1x Sham, 1x TBI, 5x Sham and 5x TBI, and the animals were anaesthetised prior to each injury (left). Experimental timeline of behavioural, RNA sequencing, biochemical and metal analyses post-TBI (right)

All mice were randomly allocated to the following experimental groups: single injury or repeat injury (total of 5 impacts) with appropriate Sham control mice in each injury group (n = 10/group equally split by sex). Repetitive injuries were delivered 48 h apart, in line with the methods described by Mouzon, et al. [43], which did not result in any unexpected mortality (Fig. 1). This study utilised one cohort of mice for all of the experiments presented in this paper, with the exception of a second cohort that was used to conduct the modified neurological severity score (mNSS) test at 9 and 12 months post-injury and a third cohort for RNA sequencing analysis at 6-months post-injury. All behavioural experiments were conducted at 1, 3, 6, 9 and 12 months post-injury, except for the Sucrose Preference Test and Morris Water Maze which were conducted at 12-months post-injury only. All biochemical and ICP-MS experiments were conducted at 12-months post-injury and RNA sequencing analysis at 6-months post-injury (Fig. 1). Experiments and analyses were conducted under blinded conditions.

### Controlled cortical impact injury

Injury induction using the controlled cortical impact (CCI) model of TBI was carried out as previously described [44]. Briefly, mild “closed-head” TBI was induced using a Hatteras PCI 3000 precision cortical impactor (Hatteras Instruments, Cary, NC). Once the mice were anesthetised using an intraperitoneal injection of 100 mg kg^− 1^ Ketamine and 10 mg kg^− 1^ of Xylazine, the head was shaved and the animals were placed in a stereotaxic frame and the head secured by ear bars. Positioned at a 20˚ angle from the midline onto the right parietal bone, the motorized 5 mm metal impactor tip delivered an injury at a 3 m/s velocity, 200 ms dwell time and a 1 mm impact depth. Immediately following delivery of the injury, the mice received an intraperitoneal injection of Buprenorphine (0.03 mg kg^− 1^) for analgesia. The mice were then immediately placed in a 37˚C heated cage for recovery and then placed back into their home cage once ambulatory. Sham control groups underwent the same procedures but did not receive the impact(s).

### Modified neurological severity score (mNSS)

The mNSS test was conducted as previously described [44]. Briefly, we incorporated the methods of Flierl, et al. [45] and Yarnell, et al. [46] in our mNSS test to evaluate neurological function (by assessing sensorimotor function, exploratory/seeking behaviour, motor coordination and balance). Our mNSS test uses a three-point Likert scale for scoring and the scoring rubric is as follows: a score of 0 was awarded if the animal completed the task, a score of 1 if the animal partially completed the task and a score of 2 if the animal failed to complete the task fully. The three-point Likert scale provides increased sensitivity for detecting subtle behavioural changes following mTBI. The test is composed of 10 tasks including the exit circle, beam walk, landing test, tail raise test, righting reflex, sound reflex, whisker stimulation task and paw flexion. Overall, the animals can reach a cumulative maximum score of 20 which is indicative of severe neurological impairment. The mNSS test was conducted at 1, 3, 6, 9 and 12 months following the last impact. All mice were acclimatised to the testing room for 30 min prior to testing. Note that the mNSS data at the 1-month time point presented in Fig. 8A and B has been previously published elsewhere [44] and that the authors have obtained permission for use of the data herein.

### Locomotor

General locomotor activity was assessed using the Locomotor (Open field) test. The mice were placed in the middle of the locomotor chamber and ambulatory time, vertical counts, zone entries and resting time were assessed over a 60-minute period using a video tracking software (Med Associates Inc). Inner and outer zones of the chambers were defined to assess the number of zone entries as a further measure of general locomotor activity. This test was conducted at 1, 3, 6, 9 and 12 months following the last impact and all mice were acclimatised to the testing room for 30 min prior to testing.

### DigiGait

The DigiGait (Mouse Specifics, Inc.) system was used to assess gait function. Once all four paws were dyed with red food colouring, each animal was positioned on top of a treadmill belt and the speed was set at 15 cm/s. The animal’s gait was recorded using a high-speed camera with each limb analysed using proprietary software and video recordings were taken for 3–4 s. This software analyses a wide variety of parameters (> 40) such as but not limited to swing duration, propulsion, stance and stride length. All animals underwent testing at 5 days pre-injury to determine baseline gait function and at 1-month following the last impact to determine the effect of single and repeat injury on gait function. The DigiGait test was conducted at 1, 3, 6, 9 and 12 months following the last impact and all mice were acclimatised to the testing room for 30 min prior to testing.

### Elevated plus maze (EPM)

The EPM test (used to assess anxiety-like behaviour) is an elevated (40 cm above the ground) perspex platform containing four arms, of which two are enclosed and two are open and the test is conducted under a low-light setting (10–20 lx) to minimise shadows. The mouse is placed facing an open arm at the centre of the maze and the time to enter each arm as well as the number of entries into each arm is recorded using a video tracking system (Topscan, Cleversys) and the mice are tested once during a 10-minute trial. Typically, mice with anxiety-like behaviour spend less time in the open arms and more time in the closed arms. The time spent in the open versus closed arms was expressed as a percentage of the total time spent in the maze. The EPM test was conducted at 1, 3, 6, 9 and 12 months post-injury and all mice were acclimatised to the testing room for 30 min prior to the start of the test. Note that the EPM data at the 1-month time point presented in Fig. 11A and B has been previously published elsewhere [44] and that the authors have obtained permission for use of the data herein.

### Sucrose preference test (SPT)

Depressive-like behaviour (specifically anhedonia) was assessed using the SPT. To allow the animals to acclimatise, the mice were individually housed in open-top cages for one week prior to the start of the test which was conducted at 12 months following the last impact. All mice were given a choice of two bottles (bottle 1 containing 1% sucrose solution in drinking water versus bottle 2 containing drinking water alone) during the first 3 days of training. The same experimenter weighed the bottles (at the same time on each day) to record consumption of each bottle. Importantly, the order of the bottles was switched each day to prevent a side preference bias. On day three, the experimenter first weighed and then removed the bottles from the cages, so that the mice were deprived of water for a maximum of 24 h. On day four (test day), the experimenter prepared fresh bottles containing 1% sucrose solution in drinking water and drinking water alone and the mice were exposed to an anxiety-provoking environment where they were placed into new open-top cages positioned under a bright LED lamp (750 lx) without food or bedding. The freshly made bottles were placed back into the cage and the mice were given 1 h to drink freely. Note that both bottles were weighed before the test and at the end of the test to determine average consumption (in grams) of each bottle. Mice experiencing anhedonia (a feature of depression) typically exhibit a lack of preference for the 1% sucrose solution in drinking water.

### Morris Water Maze (MWM)

The MWM test was used to assess spatial learning and memory function at 12 months following the last injury over an 8-day period. The pool was divided into North, East, South and West quadrants and the mice were placed into the pool in between two quadrants (at North East, South West, North West and South East). The water was made opaque by addition of a white non-toxic paint and a hidden platform was submerged 1 cm below water level located at the center of the SE quadrant. Four visual cues were placed at each of the North, East, South and West quadrants and the pool water was kept at a constant 23˚C temperature each day. A camera and tracking software (TopScanLite version 2.0) fitted above the pool recorded the time spent in each quadrant on each day. During Day 0 (acclimation day), to acclimatise the mice to the pool each mouse is given 2 × 60 s to swim freely and there is no platform in the pool at any stage. During Days 1–6 the platform was placed in the SE quadrant and each mouse was given 4 × 90 s trials, with each trial starting from a different quadrant (the order of which was randomised daily). Successful completion of the task occurred when the mouse found the hidden platform and remained on it for a minimum of 3 s. The experimenter gently guided the mouse to the platform a maximum of 3 times if the mouse did not successfully locate it. During Day 7 (Probe trial and Visual task), the platform was removed and the mice were placed into the NE quadrant once for 90 s and the time spent in each quadrant was recorded by the software. During the Probe trial, time spent in the SE quadrant is of interest to determine whether the mice have learned the location of the platform. For the Visual task, the platform was placed back into the pool above water level to test the ability of the mice to find a visible platform in 2 × 90 s trials. The test was performed by the same experimenter each day and all mice were acclimatised to the testing room for 30 min each day prior to testing.

### Y-maze

The Y-maze was used to assess short-term learning and memory. Briefly, the maze is composed of 3 arms each separated by a 120° angle which are assigned randomly as the novel, starting or familiar arm (each with visual cues). To exclude an arm-preference bias, the starting arm is randomised for each mouse prior to the start of the test. The mice are trained for 10 min and are tested for 5 min (conducted 1 h after the end of training). During training, a partition closes off the novel arm. The animals are placed into the starting arm, where they are given 10 min to explore both other arms freely. The mouse is placed back into its designated home cage at the end of the trial. One hour later, the partition closing off the novel arm is removed and the mice are placed into the same starting arm as during training and the mice are given 5 min to freely explore all three arms. A video recording system (CleverSys Inc) records the percentage time spent in each arm. The Y-maze test was conducted at 1, 3, 6, 9 and 12 months following the last impact and the mice were acclimatised to the testing room for 30 min prior to the start of the experiment.

### Perfusion and tissue processing

At 6- and 12-months post-TBI, animals were anaesthetised via an intraperitoneal injection of 80 mg kg^− 1^ of sodium pentobarbitone (Lethabarb, Virbac). The animals were perfused transcardially with phosphate buffer saline (PBS), the brains were extracted and the parietal cortex (injury site) was dissected on ice for each hemisphere (ipsilateral and contralateral). Samples were snap-frozen in dry ice and stored at − 80˚C until further use. Note that while the mice received the injuries at 3 months of age, two separate cohorts survived for a period of 6- and 12-months post-injury and are therefore referred to as 9- and 15-month-old mice hereafter.

### RNA extraction

The RNA sequencing experiment was conducted on the cohort of mice that survived for 6-months following r-mTBI only, as studies examining gene expression chronically following r-mTBI are lacking. We chose to study this at a 6-month rather than 12-month time point in the aim of capturing the dynamic and evolving genetic changes involved in neurodegenerative cascades, which we believed would be exacerbated prior to the longer term 12-month time point.

RNA was extracted from the ipsilateral and contralateral hemispheres of 5x Sham and 5x TBI parietal cortex samples using the PureLink RNA mini kit (Life Technologies, Cat# 12183018A) according to the manufacturer’s instructions. Because we were interested in extracting proteins from the same samples, this extraction protocol was adapted for Trizol purification.

Frozen samples were removed from the − 80 °C freezer and placed on dry ice. Samples were transferred to pre-chilled 2 mL round-bottom tubes containing a new 5 mm ball bearing and 1 mL of Trizol reagent (Thermo Fisher Scientific, Cat# 15596026) was added to each sample. Samples were homogenised in a TissueLyser LT (Qiagen) for 5 min at 50 Hz. Samples were further incubated with Trizol reagent for 5 min at room temperature to allow for complete dissociation of nucleoprotein complexes. 200 µL of chloroform was added, the tubes were shaken for 15 s by hand and incubated at room temperature for 3 min. Samples were centrifuged at 12,000 x g for 15 min at 4 °C. 400 µL of the colourless upper aqueous phase containing the RNA was transferred to an RNase-free 2 mL tube. Note that the interphase and organic phase were frozen on dry ice and stored at -80 °C for later use for protein isolation.

For RNA binding, 400 µL of 70% ethanol was added to obtain a final ethanol concentration of 35% and the samples were vortexed briefly. For each sample a spin cartridge was placed in a labelled collection tube and up to 700 µL of the sample was transferred to the top of the spin cartridge. Samples were centrifuged at 12,000 x g for 15 s at room temperature and the flow-through from the collection tube was discarded and the spin cartridge reinserted into the same collection tube. This process was repeated (adding 700 µL of sample and centrifuging) until the entire sample had been applied to the spin cartridge.

For DNase treatment, 350 µL of Wash Buffer I was added to the spin cartridge and centrifuged at 12,000 x g for 15 s at room temperature. The flow-through was discarded and the spin cartridge placed in a fresh collection tube. 80 µL of PureLink DNase mixture was added onto the surface of the spin cartridge membrane and incubated for 15 min at room temperature. 350 µL of Wash Buffer I was added to the spin cartridge and centrifuged at 12,000 x g for 15 s at room temperature. The collection tube was discarded and the spin column placed in a new collection tube.

For RNA collection, 350 µL of Wash Buffer II (containing ethanol) was added to the spin cartridge and centrifuged at 12,000 x g for 15 s at room temperature. Flow-through was discarded and the spin cartridge reinserted into the same collection tube. Wash Buffer II and centrifugation steps were repeated once more. The samples were then centrifuged at 12,000 x g for 2 min at room temperature to dry the membrane. Collection tubes were discarded and spin cartridges inserted into labelled recovery tubes. 100 µL of RNase-free water was added to the top/centre of the spin cartridges and incubated for 1 min at room temperature. Samples were centrifuged at 12,000 x g for 2 min at room temperature to elute the RNA from the column. Sample purity and concentration was assessed by measuring the absorbance spectra using the NanoDrop 2000 Spectrophotometer (Thermo Fisher Scientific). A260/280 ratios of our purified RNA samples were ~ 2.0. We prepared a volume of 50 µL of each sample at an RNA concentration of 100 ng/µL, as per the instructions of our sequencing provider.

### RNA sequencing

cDNA library preparation, quality control (QC) via Tapestation and bulk RNA sequencing (RNA-Seq) was performed by the Australian Genome Research Facility (AGRF) in Melbourne, Australia using the PerkinElmer LabChip GX. The lowest RNA quality score (RQS) was 7.9 and samples with RQS ≥ 7 are considered high quality. cDNA library preparation was set at 20 M reads for mouse samples. RNA samples were sequenced as 100 bp, single-end sequencing reads.

### Differential gene expression (DGE) analysis

Alignment was performed on Spartan, a High Performance Computing (HPC) system operated by Research Computing Services at The University of Melbourne. Snakemake was used as a workflow management tool to generate a workflow for alignment of the reads to a reference genome [47]. Briefly, the raw sequencing reads (FASTQ files) for each sample were aligned to the GRCm39 mouse reference genome (retrieved from https://www.ncbi.nlm.nih.gov/assembly/GCF_000001635.27) using the splice-aware STAR aligner [48]. Trimming of adapter sequences was performed and quality control of the alignment was assessed via FastQC [49] and MultiQC [50] reports. The code for reproducing this part of the analysis is named “Snakefile” and has been added to GitHub (https://github.com/sydneyjuan/RNAseq_r-mTBI/blob/main/Snakefile).

For DGE analysis, the output files generated from the alignment were used as input files into the R software [51] and a gene counts table for all 40 samples was created. The gene counts table (https://github.com/sydneyjuan/RNAseq_r-mTBI/blob/main/New_Gene_Counts.txt) and sample information (https://github.com/sydneyjuan/RNAseq_r-mTBI/blob/main/Sample_Info.txt) text files were read into R. The gene counts table was converted to a DGEList object using the edgeR package [52] and genes were annotated using the org.Mm.eg.db package [53]. Counts per million (CPM) values were generated from the gene counts table, which normalises for the different sequencing depths of each sample and lowly expressed genes were then filtered out. We produced a heatmap of the top 50 most variable genes using the NMF package [54]. A design matrix (2 × 2 factorial design) was defined with the factors being treatment group (5x Sham and 5x TBI animals), sex (males and females) and hemisphere (ipsilateral and contralateral). Differential expression analysis was performed via limma-voom [55]. The code for reproducing this part of the analysis is named “r-mTBI_DGEanalysis_SJ” and has been added to GitHub (https://github.com/sydneyjuan/RNAseq_r-mTBI/blob/main/r-mTBI_DGEanalysis_SJ.Rmd).

### Biochemistry

#### Sample preparation

At 12-months post-injury, parietal cortex samples from both injury groups were homogenized (1:10 w/v) in homogenisation buffer containing ice-cold phosphate buffered saline (PBS), Roche complete EDTA-free Protease Inhibitor Cocktail tablets (Sigma Aldrich) and Roche Phosphatase (PhosSTOP) Inhibitor tablets (Sigma Aldrich) and were sonicated (at 30% amplitude) using an ultrasonic liquid processor (Sonicator® Misonix). The samples (100 µL) were fractionated into soluble and insoluble fractions by centrifugation at 100,000 x g at 4˚C for 30 min. The supernatant was collected as the soluble fraction. The remaining pellet was re-suspended into 100 µL of homogenisation buffer and collected as the insoluble fraction.

### Western blot

Western blots were conducted as previously described with some modifications [44]. Protein concentrations were determined via Pierce BCA protein assay (Life Technologies) so that samples were loaded equally onto the gels (10 µg of protein for ferroportin and transferrin receptor (TfR) antibodies, 20 µg for DMT1, and 5 µg for the rest of the antibodies). Samples were prepared for SDS-PAGE by the addition of 4x Bolt™ LDS sample buffer (Life Technologies) and 50x Bond Breaker TCEP reducing agent (Life Technologies) to a final 1x concentration. Samples were heated at 90˚C for 5 min, loaded onto 17-well Bolt™ 4–12% Bis-Tris Plus Gels (Life Technologies) with the Odyssey One-Colour Protein molecular weight marker (Millennium Science) and run for 1 h at 125 V in 1x MES buffer (Life Technologies). Gels were transferred to Immobilon-FL PVDF membranes (Merck Millipore) using the Mini Blot Module wet-transfer device (Invitrogen) and run at 15 V for 1 h in 1x Bolt™ transfer buffer (Life Technologies). Membranes were incubated with 5 mL of Licor Revert Total Protein Stain (Millennium Science) as per the manufacturer’s instructions. Using a LI-COR Odyssey Imaging system (LI-COR Biosciences, USA), the membranes were imaged for 2 min in the 700 nm channel. Membranes were blocked with 5% skim milk powder (omitted when targeting phosphorylated proteins) and 1% BSA in 1x Tris-buffered saline with tween 20 (TBST; 10 mM Tris, 150 mM NaCl, 0.1% Tween 20) for 30 min at room temperature and then incubated with primary antibody (Table 1) diluted in 3% BSA in 1x TBST overnight at 4˚C. Membranes were rinsed with 1x TBST and incubated with the appropriate IRDye 680 (red) or 800 (green) secondary antibody (Millennium Science) diluted in 0.01% SDS in 1x TBST for 30 min at room temperature. For ferroportin and transferrin receptor, membranes were incubated with secondary antibody for 1 h at room temperature. Membranes were washed and imaged using a LI-COR Odyssey Imaging system (LI-COR Biosciences, USA) and analysed using Image Studio Lite software (LI-COR Biosciences, Lincoln NE, USA). All samples were first normalised to total protein content (Revert) and then a between blot normalisation was conducted using a blot control sample present on each membrane. In addition, phospho-tau proteins were normalised to total tau levels and phosphorylated GSK3β levels were normalised to total GSK3β levels to determine the ratio of phosphorylated/total GSK3β.

**Table 1:** List of primary antibodies used for western blots

Antibody	Species	Dilution	Company	Catalogue number
Total tau	Rabbit	1:1000	Agilent Technologies	A002401-2
Phospho-Tau (Ser396)	Mouse	1:1000	Thermo Fisher	35-5300
Anti-Tau oligomer (T22)	Rabbit	1:1000	Merck	ABN454
Recombinant Anti-Tau (phospho T231)	Rabbit	1:1000	Abcam	ab151559
Phospho-Tau (Ser202)	Rabbit	1:1000	Cell Signaling Technology	39,357 S
Anti-APP (22C11)	Mouse	1:1000	Konrad Beyreuther - University of Heidelberg, 22C11	22C11
PP2A A subunit	Rabbit	1:1000	Cell Signaling Technology	2041
PP2A B subunit	Rabbit	1:1000	Cell Signaling Technology	2290
PP2A C subunit	Rabbit	1:1000	Cell Signaling Technology	2259
GSK3β	Rabbit	1:1000	Cell Signaling Technology	9315
p-GSK3β	Rabbit	1:1000	Cell Signaling Technology	9336
Pin-1	Rabbit	1:1000	Cell Signaling Technology	3722
PME-1	Rabbit	1:500	Thermo Fisher	PA5-27754
Ferritin	Rabbit	1:1000	Abcam	ab75973
Ferroportin	Rabbit	1:1000	Novus Biologicals	NOVNBP121502
Transferrin receptor	Mouse	1:2000	Thermo Fisher	13-6800
DMT1	Mouse	1:500	Abcam	ab55735

### Metal analysis

At 12-months post-injury, brain metal analysis was performed in both injury groups using the soluble and insoluble brain homogenates described in the sample preparation section. Brain metal content was measured in ipsilateral and contralateral homogenized parietal cortex samples.

### Inductively coupled plasma-mass spectrometry (ICP-MS)

ICP-MS was performed as previously described [56]. Briefly, ipsilateral and contralateral cortical brain homogenates were lyophilized and the dry material was digested with 50 µL of Nitric Acid (HNO_3_, 65% Suprapur, Merck) overnight. The samples were further digested by heating to 90^°^C for 20 min using a heating block. Samples were removed from the heating block and an equivalent volume of 50 µL of Hydrogen Peroxide (H_2_O_2_, 30% Aristar, BDH) was added to each sample. Samples were allowed to stop digesting for ~ 30 min before heating again for 15 min at 70^°^C. The average reduced volume was determined and the samples further diluted with 1% HNO_3_. All measurements were performed on an Agilent 7700 Series ICP-MS instrument (Agilent Technologies, CA, USA) under routine multi-element operating conditions using a Helium Reaction Gas Cell. The instrument was calibrated with 0, 5, 10, 50, 100, and 500 ppb of certified multi-element ICP-MS standard calibration solutions (ICP-MS-CAL2-1, ICP-MS-CAL-3 and ICP-MS-CAL-4, Accustandard, CT, USA) for a range of elements. A certified internal standard solution containing 200 ppb of Yttrium (Y89) was used as the internal control (ICP-MS-IS-MIX1-1, Accustandard). Metal content (µg/g) was normalised to protein concentration which was determined using the BCA Protein Assay Kit (Thermo Fisher Scientific).

### Statistical analysis

Two separate cohorts were used for the mNSS (one each for 1–6 months and 9–12 months post-injury). The reason for this was as follows; we had originally used the standard NSS test [45] on the second cohort of mice surviving for 12-months post-injury at the 1, 3 and 6 month post-injury time points. Upon analysis of that data (performed by an experimenter that was blinded to treatment group and injury group) the findings revealed no statistical differences. Because the standard NSS test is a gold standard test traditionally used in severe TBI models, we suspected that it may not be sensitive enough (largely due to its pass/fail scoring system) to detect more subtle changes in neurological function following a mild injury. Therefore, we incorporated the methods of Flierl, et al. [45] for the standard NSS and Yarnell, et al. [46] for the revised NSS (NSS-R), which uses a three-point Likert scale and has increased sensitivity for detecting subtle changes in neurological function, in our mNSS test. As such, the mNSS test was performed on the 12-month cohort at the 9–12 month time points and on the 6-month cohort at the 1–6 month time points. Because both cohorts followed similar trends, the data are presented on the same graph solely for illustrative purposes, with the two cohorts represented by a gap on the x-axis in the relevant figure. For the 5 impact group at the 1–6 month time points, a Two-way repeated measures ANOVA was used to determine main effects and interaction between factors with a Holm-Sidak post hoc test to correct for multiple comparisons. For the 9–12 month time points, a mixed-effects analysis was used to account for missing values followed by a Holm-Sidak post hoc test. For the 1 impact group, a Two-way repeated measures ANOVA with Holm-Sidak post hoc test was used for all time points.

For the Locomotor test, a mixed-effects analysis was used to account for missing values followed by a Holm-Sidak post hoc test when appropriate for all parameters assessed for the 1x and 5x injury groups. Because zone entries and resting time did not have missing values in the 1x injury group, these were analysed by Two-way repeated measures ANOVA. For the EPM and DigiGait, a mixed-effects analysis was used to account for missing values followed by an Uncorrected Fisher’s LSD test which does not correct for multiple comparisons. The reasoning behind this are two fold, the first is that repeated testing is generally not recommended for both of these tests, and second is that the nature of these tests means that variation increases every time the test is conducted. Therefore, each comparison stands alone. For the Y-maze data, a mixed-effects analysis with Holm-Sidak post hoc test was used when appropriate. For the MWM data, a Two-way repeated measures ANOVA with Holm-Sidak post hoc test was used to assess differences in average trial duration for the 5 hit group. For the 1 hit group, a mixed-effects analysis was used to account for missing values. Unpaired two-tailed Student’s t-tests were used to assess differences between two groups for the Probe trial of the MWM, the western blot and the ICP-MS data. For the SPT test, an ordinary Two-way ANOVA was used with Holm-Sidak post hoc test. Data are expressed as mean ± SEM. All statistical analyses were performed using GraphPad Prism version 8 and a Grubbs’ test was used to identify any outliers. *P* < 0.05 was considered statistically significant. Lastly, DGE analysis was performed using RStudio version 1.1.463. Genes that had a false discovery rate (FDR) less than 5% were considered statistically significant.

## Results

### No differentially expressed genes (DEGs) at 6-months post r-mTBI

**Fig. 2:**
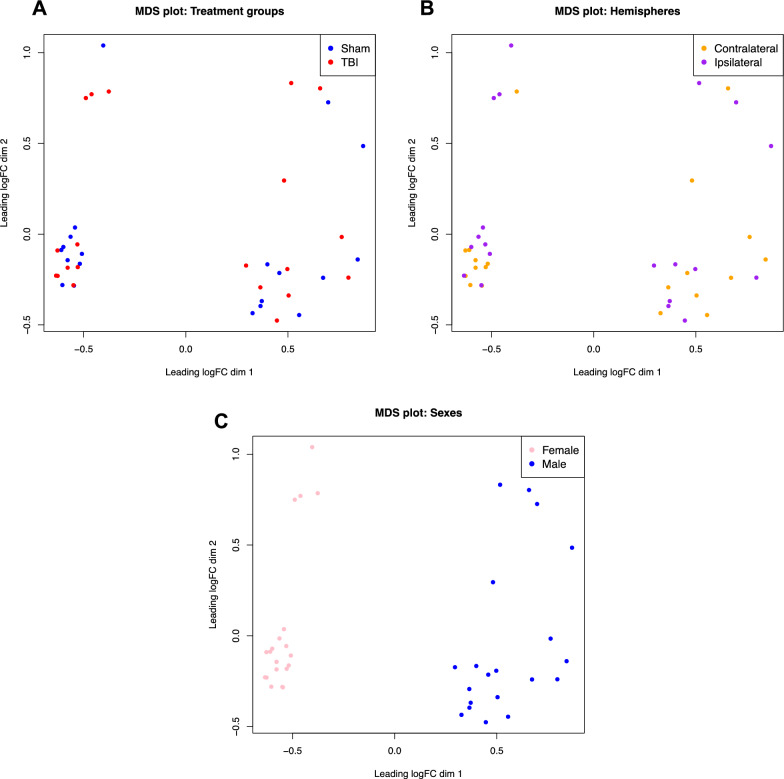
Multidimensional scaling (MDS) plots for each variable. **A** MDS plot of 5x Sham and 5x TBI animals revealed no clustering of samples by treatment group. **B** MDS plot of ipsilateral and contralateral hemispheres revealed no clustering of samples by hemisphere. **C** MDS plot of male and female animals revealed clustering of samples by sex. n = 10 Sham, 10 TBI (5 of each sex per treatment group for both hemispheres, total n = 40 samples)

Following bulk RNA sequencing, we performed a DGE analysis to determine whether r-mTBI would induce changes in gene expression typically involved in neurodegenerative processes at 6-months post-injury. After filtering for lowly expressed genes, we identified 17,517 genes with measured expression in our dataset. We generated multidimensional scaling (MDS) plots to examine the greatest source of variation in the data. Surprisingly, samples did not cluster by treatment group (Fig. 2A) or by hemisphere (Fig. 2B) but samples did cluster by sex (Fig. 2C).

**Fig. 3:**
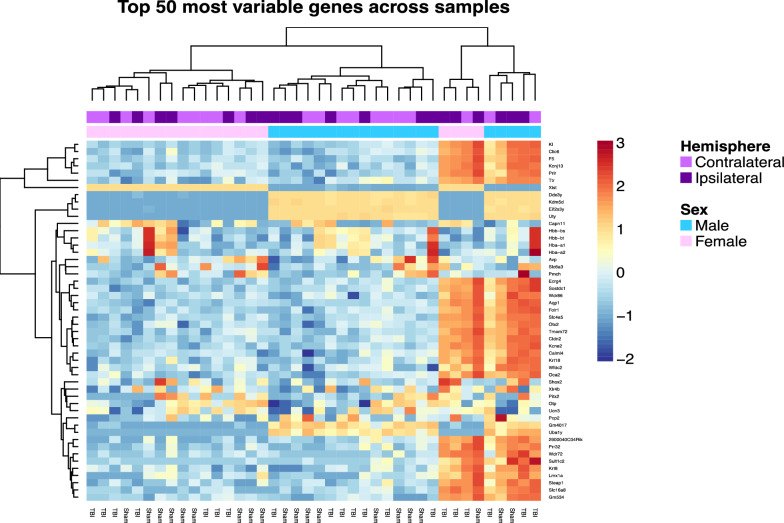
Heatmap of hierarchical clustering of samplesfor the top 50 most variable genes in the dataset. The colour key represents Z scores, where a value of 0 indicates average gene expression, -2 indicates underexpression and + 3 indicates overexpression. This heatmap further demonstrates clustering of genes by sex

We then generated a heatmap to examine hierarchical clustering of our samples for the top 50 most variable genes (Fig. 3). The heatmap further demonstrated the previously observed clustering of certain genes by the sex variable.

**Fig. 4:**
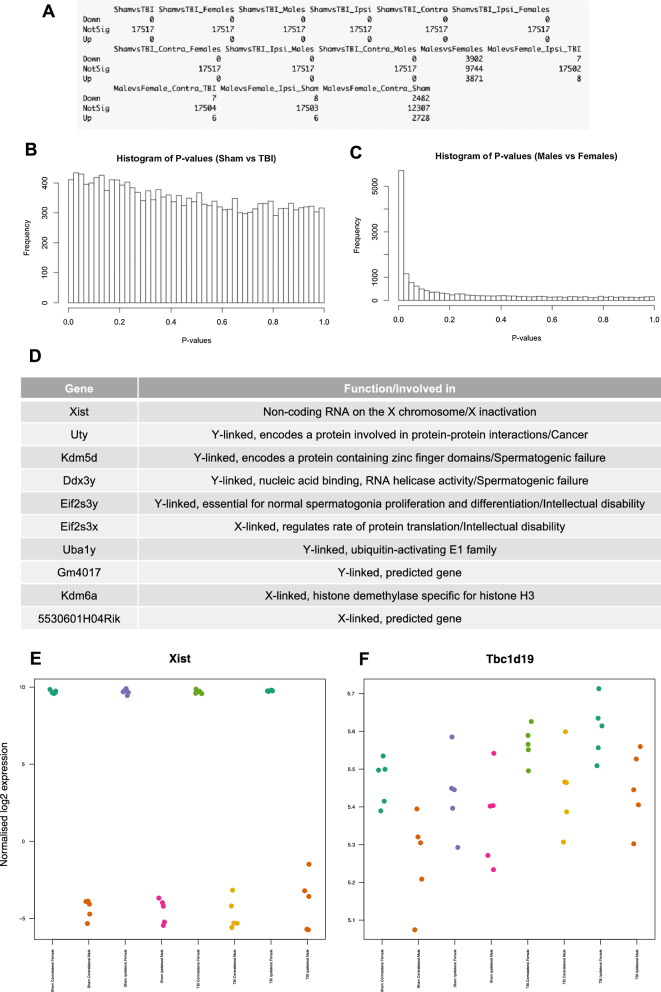
Differential gene expression (DGE) analysis. **A** Table as outputted from RStudio of the comparisons made for DGE analysis. Sham versus TBI comparisons yielded no differentially expressed genes, but the Male versus Female comparisons yielded a number of differentially expressed genes. **B** Histogram of P-values exhibit a uniform distribution for the Sham vs. TBI comparison, indicating no significant difference. **C** Histogram of P-values have a higher frequency at smaller significant P-values and a lower frequency at higher non-significant P-values for the Male vs. Female comparison, indicating a significant difference. **D** List of the 10 most differentially expressed genes from the Males versus Females comparison, all genes are located on sex chromosomes. **E** Stripchart of normalised log2 expression levels of individual samples for the most differentially expressed “Xist” gene (significant) in Males vs. Females, which is located on the X chromosome. **F** Stripchart of normalised log2 expression levels of individual samples for the most differentially expressed “Tbc1d19” gene (not significant) in Sham vs. TBI, which is a protein-coding gene located on chromosome 4 that is predicted to act as a GTPase activating protein. Plots in **B, C, E** and **F** were generated in RStudio

Despite these findings, we created a design matrix for the groups and tested for differential gene expression and we adjusted for multiple comparisons by using the Benjamini & Hochberg “BH” method, also known as the FDR [57]. We found no DEGs in any of the 9 Sham vs. TBI comparisons, however there were a number of DEGs in the “MalesvsFemales” and “MalevsFemale_Contra_Sham” comparisons (Fig. 4A). To confirm these findings we generated histograms of the entire range of P-values (0–1) for the Sham vs. TBI and Males vs. Females comparisons (Fig. 4B and C). In the Sham vs. TBI comparison, P-values were uniformly distributed across the entire range of P-values, confirming no significant differences (i.e. no DEGs) in this comparison. However in the Males vs. Females comparisons, the frequency of P-values was very high for the small, significant range of P-values, and the frequency dropped off at higher, non-significant P-values, confirming a significant difference (i.e. DEGs) in this comparison. Because we were testing the hypothesis that r-mTBI would lead to changes in gene expression, then the lack of changes between treatment groups (TBI and Sham) was unexpected. We identified the top 10 most DEGs between Males and Females and unsurprisingly found that all 10 genes were located on sex chromosomes (Fig. 4D). Finally, we generated a stripchart of the most DEGs for each comparison (Fig. 4E for Sham vs. TBI and Fig. 4F for Males vs. Females), which allowed us to visualise the normalised log2 expression level of individual samples for each gene. The Xist gene in the Males vs. Females comparison was highly expressed in female mice (Fig. 4E), whereas the Tbc1d19 gene, which is predicted to act as a GTPase activating protein, was similarly expressed between Sham and TBI mice (Fig. 4F). Overall, our RNA sequencing experiment yielded no DEGs as a function of r-mTBI, and further analysis of male and female mice separately will be required to elucidate whether r-mTBI can induce gene expression changes at this chronic 6-month timeframe.

#### Changes in brain iron metabolism following r-mTBI

**Fig. 5:**
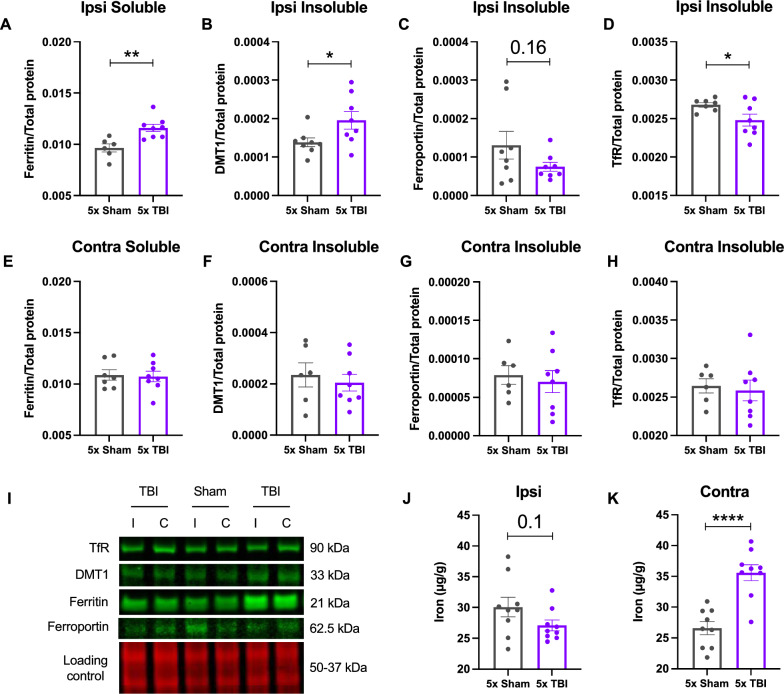
Expression of iron-regulatory proteins and total iron content in mice following single or r-mTBI. Iron-regulatory proteins (**A-I**) and total iron content (**J** and **K**) in the ipsilateral and contralateral hemispheres of the parietal cortex (injury site) following five impacts at 12 months post-injury. Iron-regulatory proteins were measured by western blot and total iron content via ICP-MS. **(A)** Significant increase in ferritin in the ipsilateral soluble fraction of 5x TBI animals compared to Shams. **B** Significant increase in DMT1, **C** trend towards a decrease in ferroportin and **D** significant decrease in transferrin receptor (TfR) in the ipsilateral insoluble fraction of 5x TBI animals compared to Shams, respectively. **E** No significant differences in ferritin in the contralateral soluble fraction of 5x TBI animals compared to Shams. No significant differences in DMT1 **(F)**, ferroportin **G** or transferrin receptor **H** in the contralateral insoluble fraction of 5x TBI animals compared to Shams. **I** Representative western blot images correspond to the hemisphere and fraction of the protein depicted in the graph. Example of a loading control blot for ferritin from the 5 hit group. **J** ICP-MS revealed no changes in total iron content in the ipsilateral hemisphere of 5x TBI animals compared to Shams. **K** Significant increase in total iron content within the contralateral hemisphere of 5x TBI animals compared to Shams. Note that each of the antibodies were run on separate blots but are represented herein as a composite image for the purpose of clarity. Unpaired two-tailed Student’s t-test. Data expressed as mean ± SEM, *P < 0.05, **P < 0.01, ****P < 0.0001, n = 6–9 (Sham), n = 8–9 (TBI)

We investigated whether there were changes in the proteins that regulate iron levels within the brain, specifically transferrin receptor (TfR) (90 kDa), DMT1 (33 kDa), ferritin (21 kDa) and ferroportin (62.5 kDa) in the soluble and insoluble fractions of the ipsilateral and contralateral hemispheres of the parietal cortex (injury site) at 12 months post-injury. Figure 5 depicts each protein of interest once for a particular fraction and hemisphere. Using western blots, we found that there was a significant increase in ferritin levels (*t*_12_ = 3.581, *P* = 0.0038) in the ipsilateral soluble fraction of 5x TBI animals compared to Shams (Fig. 5A). There was also a significant increase in DMT1 (*t*_14_ = 2.250, *P* = 0.0411), a significant decrease in TfR (*t*_13_ = 2.248, *P* = 0.0425) and a trend towards decreased ferroportin levels (*t*_14_ = 1.481, *P* = 0.1607) in the ipsilateral insoluble fraction of 5x TBI animals compared to Shams (Fig. 5B, D and C respectively). There were no significant changes in ferritin, DMT1, ferroportin or TfR in the contralateral hemisphere following five impacts (Fig. 5E-H), or in either hemisphere following a single impact (data not shown). Because of these iron-regulatory protein changes, it was important to then determine whether there were alterations in total iron content. Using ICP-MS we found a slight trend towards decreased total iron content in the ipsilateral hemisphere of the parietal cortex in 5x TBI animals versus Shams (*t*_16_ = 1.643, *P* = 0.1199, Fig. 5J). However, there was a significant increase in total iron content (*t*_16_ = 5.400, *P* < 0.0001) in the contralateral hemisphere of 5x TBI animals versus Shams (Fig. 5K). Additionally, ICP-MS also revealed a significant increase in total zinc (*t*_16_ = 4.777, *P* = 0.0002) and copper (*t*_16_ = 4.350, *P* = 0.0005) levels in the contralateral hemisphere following five impacts compared to Shams (Additional file 1: Fig. S1C and D, respectively). There were no changes in total zinc or copper levels in the ipsilateral hemisphere following five impacts (Additional file 1: Fig. S1A and B, respectively). Following a single injury, there was a significant decrease in zinc (*t*_17_ = 2.425, *P* = 0.0267) and a significant increase in copper (*t*_18_ = 2.948, *P* = 0.0086) but no changes in iron in the ipsilateral hemisphere (Additional file 1: Fig. S1B, C and A, respectively). There were no changes in all three metals in the contralateral hemisphere (Additional file 1: Fig. S1D, E and F).

#### Tau phosphorylation is not altered at 12-months following single or r-mTBI

**Fig. 6:**
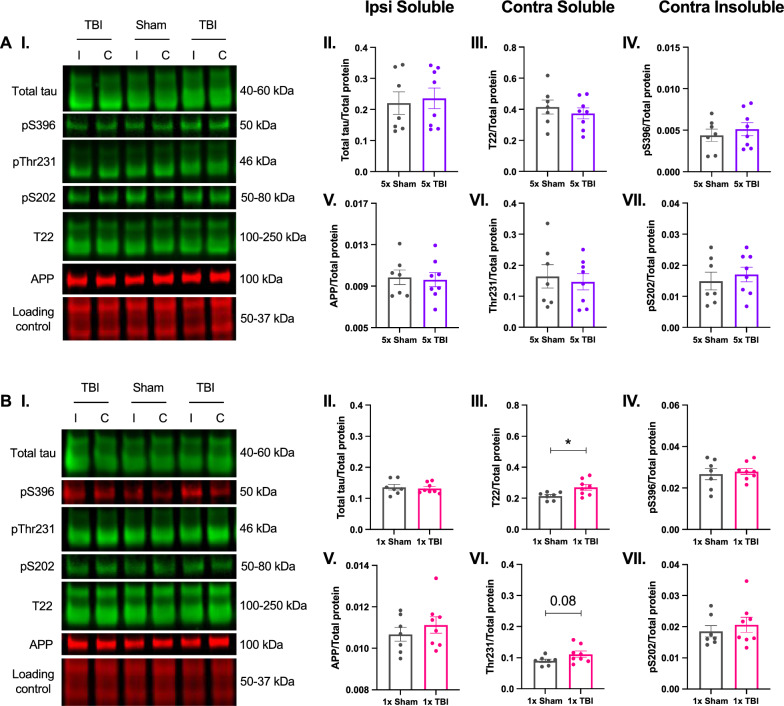
Expression profile of neurodegeneration-associated proteins in mice following single or r-mTBI. Western blots in the parietal cortex following five (**A**) and one (**B**) impact at 12 months post-injury. **A-I** to **A-VII** Following five impacts, there were no significant changes in the expression of any proteins of interest at 12 months post-injury. **B-III** Following one impact, there was a significant increase in T22 oligomeric tau and (**B-VI**) a trend towards increased Thr231 in the soluble fraction of the contralateral hemisphere at 12 months post-injury. **B-II, IV, V** and **VII** There were no other changes in the expression of other proteins of interest at 12 months post-injury. Representative western blot images (**A-I** and **B-I**) correspond to the hemisphere and fraction of the protein depicted in the graph. Example of a loading control blot for Total tau from the 5 hit group (**A-I**) and the 1 hit group (**B-I**). All phospho-tau proteins are normalised to total protein content and total tau of each sample. Note that each of the antibodies were run on separate blots but are represented herein as a composite image for the purpose of clarity. Unpaired two-tailed Student’s t-test. Data expressed as mean ± SEM, *P < 0.05, n = 7 (Sham), n = 8 (TBI)

We also investigated whether there were changes in the expression of proteins involved in neurodegenerative processes, specifically the levels of Total tau (40–60 kDa), pSer396 (50–55 kDa), T22 oligomeric tau (100–250 kDa), pThr231 (46 kDa), pSer202 (50–80 kDa) and amyloid precursor protein (APP, 100 kDa) in the soluble and insoluble fractions of the ipsilateral and contralateral hemispheres of the parietal cortex (injury site) at 12 months post-injury. Figure 6 below depicts each protein of interest for a particular fraction and hemisphere, therefore note that only a subset of the data are shown for clarity. Surprisingly, following five impacts, we found no significant changes in the expression of Total tau (*t*_13_ = 0.3179, *P* = 0.7556, Fig. 6A-II) and APP (*t*_13_ = 0.2379, *P* = 0.8157, Fig. 6A-V) in the soluble fraction of the ipsilateral hemisphere. We found no significant differences in T22 oligomeric tau (*t*_13_ = 0.7335, *P* = 0.4763, Fig. 6A-III) or Thr231 (*t*_13_ = 0.3894, *P* = 0.7033, Fig. 6A-VI) in the soluble fraction of the contralateral hemisphere. Lastly, there were no changes in the levels of pS396 (*t*_13_ = 0.6709, *P* = 0.5140, Fig. 6A-IV) or pS202 (*t*_13_ = 0.5781, *P* = 0.5731, Fig. 6A-VII) in the insoluble fraction of the contralateral hemisphere. Following one impact, there were no significant changes in the levels of Total tau (*t*_13_ = 0.3672, *P* = 0.7194, Fig. 6B-II) or APP (*t*_13_ = 0.8552, *P* = 0.4079, Fig. 6B-V) in the ipsilateral soluble fraction. However, there was a significant increase in T22 oligomeric tau (*t*_13_ = 2.597, *P* = 0.0221, Fig. 6B-III) and a trend towards increased Thr231 (*t*_13_ = 1.850, *P* = 0.0871, Fig. 6B-VI) in the contralateral soluble fraction at 12 months post-injury. Lastly, there were no changes in the levels of pS396 (*t*_13_ = 0.4243, *P* = 0.6783, Fig. 6B-IV) or pS202 (*t*_13_ = 0.6814, *P* = 0.5076, Fig. 6B-VII) in the contralateral insoluble fraction at 12 months post-injury. Collectively, these data demonstrate that tau pathology is not present at 12 months following five impacts. On the other hand, tau pathology is an inconsistent feature at 12 months after receiving a single impact. These results bring to question the role and relevance of tau in the chronic stage of r-mTBI.

#### No changes in tau-regulatory proteins at 12-months post single or r-mTBI

**Fig. 7:**
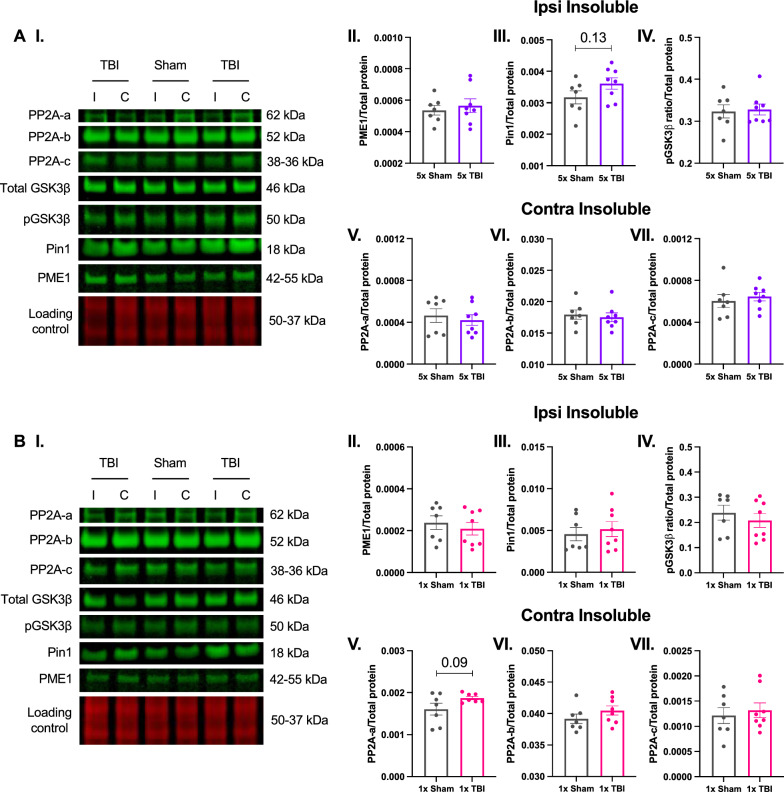
Expression of iron-regulatory proteins and total iron content in mice following single or r-mTBI. Iron-regulatory proteins (**A-I**) and total iron content (**J** and **K**) in the ipsilateral and contralateral hemispheres of the parietal cortex at 12 months post-injury. (**A-II** to **VII**) Following five impacts, there were no significant changes in the expression of any proteins of interest at 12 months post-injury despite a slight trend towards increased Pin1 in the ipsilateral insoluble fraction (**A-III**). **B-II** to **VII** Following one impact, there were no significant changes in the expression of any proteins at 12 months post-injury, despite a trend towards increased levels of PP2A-a subunit (**B-V**) in the contralateral insoluble fraction. Representative western blot images (**A-I** and **B-I**) correspond to the hemisphere and fraction of the protein depicted in the graph. Example of a loading control blot for PME1 from the 5 hit group (**A-I**) and the 1 hit group (**B-I**). Note that each of the antibodies were run on separate blots but are represented herein as a composite image for the purpose of clarity. Unpaired two-tailed Student’s t-test. Data expressed as mean ± SEM, *P < 0.05, n = 7 (Sham), n = 7–8 (TBI)

We also investigated whether there were changes in the proteins that regulate tau phosphorylation specifically kinase and phosphatase proteins, therefore we examined the expression of PP2A-a subunit (62 kDa), PP2A-b subunit (52 kDa), PP2A-c subunit (38 − 36 kDa), PME1 (42–55 kDa), Pin1 (18 kDa), Total GSK3β (46 kDa) and Phospho-GSK3β (50 kDa) in the soluble and insoluble fractions of the ipsilateral and contralateral hemispheres of the parietal cortex (injury site) at 12 months post-injury. Figure 7 depicts each protein of interest once for a particular fraction and hemisphere. Following five impacts, there were no significant differences in PME1 (*t*_13_ = 0.5410, *P* = 0.5977, Fig. 7A-II) or the ratio of Phospho/Total GSK3β (*t*_13_ = 0.2377, *P* = 0.8158, Fig. 7A-IV), however there was a slight trend towards increased Pin1 (*t*_13_ = 1.616, *P* = 0.1302, Fig. 7A-III) in the insoluble fraction of the ipsilateral hemisphere. Similarly, there were no significant changes in the levels of PP2A-a Subunit (*t*_13_ = 0.5307, *P* = 0.6046, Fig. 7A-V), b Subunit (*t*_13_ = 0.4249, *P* = 0.6778, Fig. 7A-VI) or c Subunit (*t*_13_ = 0.5803, *P* = 0.5716, Fig. 7A-VII) in the insoluble fraction of the contralateral hemisphere at 12 months post-injury. Following one impact, we found no significant differences in the levels of PME1 (*t*_13_ = 0.6450, *P* = 0.5302, Fig. 7B-II), Pin1 (*t*_13_ = 0.4848, *P* = 0.6359, Fig. 7B-III) or the ratio of Phospho/Total GSK3β (*t*_13_ = 0.7728, *P* = 0.4535, Fig. 7B-IV) in the insoluble fraction of the ipsilateral hemisphere. However, there was a trend towards increased levels of PP2A-a subunit (*t*_12_ = 1.812, *P* = 0.0950, Fig. 7B-V), but no differences in PP2A-b subunit (*t*_13_ = 1.265, *P* = 0.2280, Fig. 7B-VI) or c subunit (*t*_13_ = 0.4885, *P* = 0.6333, Fig. 7B-VII) in the insoluble fraction of the contralateral hemisphere at 12 months post-injury. No other changes were seen for proteins of interest in other hemispheres and fractions not presented here. Collectively, this data demonstrates that tau-regulatory proteins are not altered at 12 months following either a single impact or five impacts.

### Neurological assessment following a single or r-mTBI

**Fig. 8:**
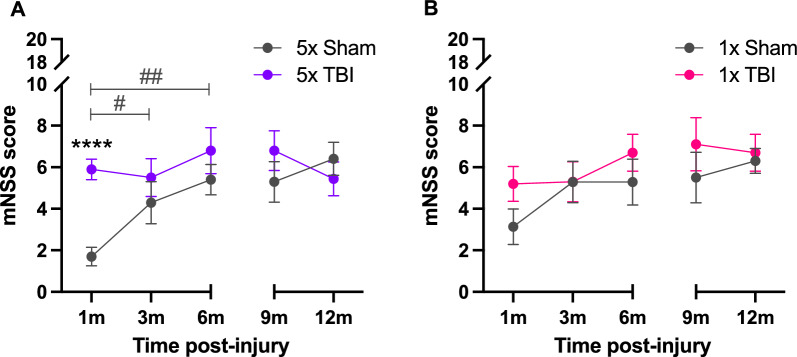
Neurological status in mice following single or r-mTBI. Assessment of neurological status using the mNSS test following five (**A**) and one (**B**) impact at 1, 3, 6, 9 and 12 months post-injury. **(A)** Significant increase in mNSS scores following five impacts compared to Shams at 1-month post-injury, with no differences thereafter. **(B)** No significant differences in mNSS scores following one impact across all time points. Two separate cohorts of animals were used at 1–6 and 9–12 months post-injury for both injury groups, represented by a gap on the x-axis. A Two-way repeated measures ANOVA (1–6 months) and a mixed-effects analysis (9–12 months) were conducted separately for each cohort. Because both cohorts followed similar trends, the data are presented on the same graph solely for illustrative purposes. Note that the 1-month time point presented in this figure has been previously published elsewhere [44] and that the authors have obtained permission to re-use this data. Data expressed as mean ± SEM, #*P* < 0.05, ##*p* < 0.01, ****P < 0.0001, n = 7–10 (Sham), n = 9–10 (TBI), * indicates a significant difference between Sham and TBI animals, # indicates a significant difference within Sham animals across time points

We evaluated neurological function using the mNSS at 1, 3, 6, 9 and 12 months following the last impact. From 1 to 6 months following five impacts, there was a significant effect of time post-injury (*F*_1.774,31.94_ = 6.924, *P* = 0.0043), treatment group (*F*_1,18_ = 6.031, *P* = 0.0244), subject (*F*_18,36_ = 3.343, *P* = 0.0010) and a significant time post-injury x treatment group interaction effect (*F*_2,36_ = 3.680, *P* = 0.0351) (Fig. 8A). Post hoc analysis revealed a significant increase in mNSS scores in the 5x TBI group at 1-month post-injury compared to Shams (*t*_17.76_ = 6.223, *P* < 0.0001). There were no significant differences in mNSS scores at 3- or 6-months post-injury following five impacts, despite these scores being higher in the 5x TBI group compared to Shams. There was however a significant increase in mNSS scores within the Sham group from 1 to 3 months (*t*_9_ = 3.027, *P* = 0.0284) and 1 to 6 months (*t*_9_ = 4.772, *P* = 0.0030) post-injury therefore reaching similar mNSS scores as the 5x TBI mice by 6 months (Fig. 8A). There were no significant differences in mNSS scores at 9 and 12 months following five impacts (Fig. 8A). From 1 to 6 months following one impact, there was a significant effect of subject (*F*_15,30_ = 3.181, *P* = 0.0034), however post hoc analysis revealed no significant differences between treatment groups across time (Fig. 8B). Similarly, from 9 to 12 months following one impact there was a significant effect of subject (*F*_18,18_ = 5.219, *P* = 0.0005) but post hoc analysis revealed no differences between groups (Fig. 8B). Unsurprisingly, mNSS scores for Sham animals in both the 1x and 5x injury groups progressively increased over time, which is expected in healthy ageing. In both injury groups, mNSS scores for TBI animals were initially higher on average from 1 to 9 months post-injury, but by 12 months post-injury mNSS scores for TBI animals were no different to their Sham counterparts. This suggests that deficits in neurological function, which we have previously shown to occur in young-adult (4-month-old) mice at a sub-acute (1-month) time point following five impacts [44], do not extend to chronic stages post-injury. Importantly this demonstrates that deficits in neurological function are mild, and that by 6–12 months post-injury the deficit between Sham and TBI animals is similar.

### Locomotor activity is increased following repeated but not a single injury

We assessed general locomotor activity by examining ambulatory time, zone entries, resting time and vertical counts between treatment groups. For ambulatory time following five impacts, there was no treatment group or interaction effect however time post-injury showed a strong trend for significance (*F*_3.067,54.44_ = 2.620, *P* = 0.0588, Fig. 10A). Following one impact, there was a significant time post-injury effect (*F*_2.712,45.43_ = 4.243, *P* = 0.0122) but no treatment group (*F*_1,18_ = 0.6028, *P* = 0.4476) or interaction effect (*F*_4,67_ = 0.6224, *P* = 0.6481) (Fig. 10B). Post hoc analysis revealed no significant differences in ambulatory time between 1x TBI animals and Shams. We note that 5x TBI animals spent on average more time being ambulatory than Shams (not significant) at 9 and 12-month time points. This was further highlighted by the significant decrease in ambulatory time from 6 to 12 months post-injury in the Sham group, which was not reflected following five impacts. Interestingly, the reverse was true for the 1x injury group, with 1x TBI animals spending on average slightly less time being ambulatory compared to Shams (not significant).

**Fig. 9:**
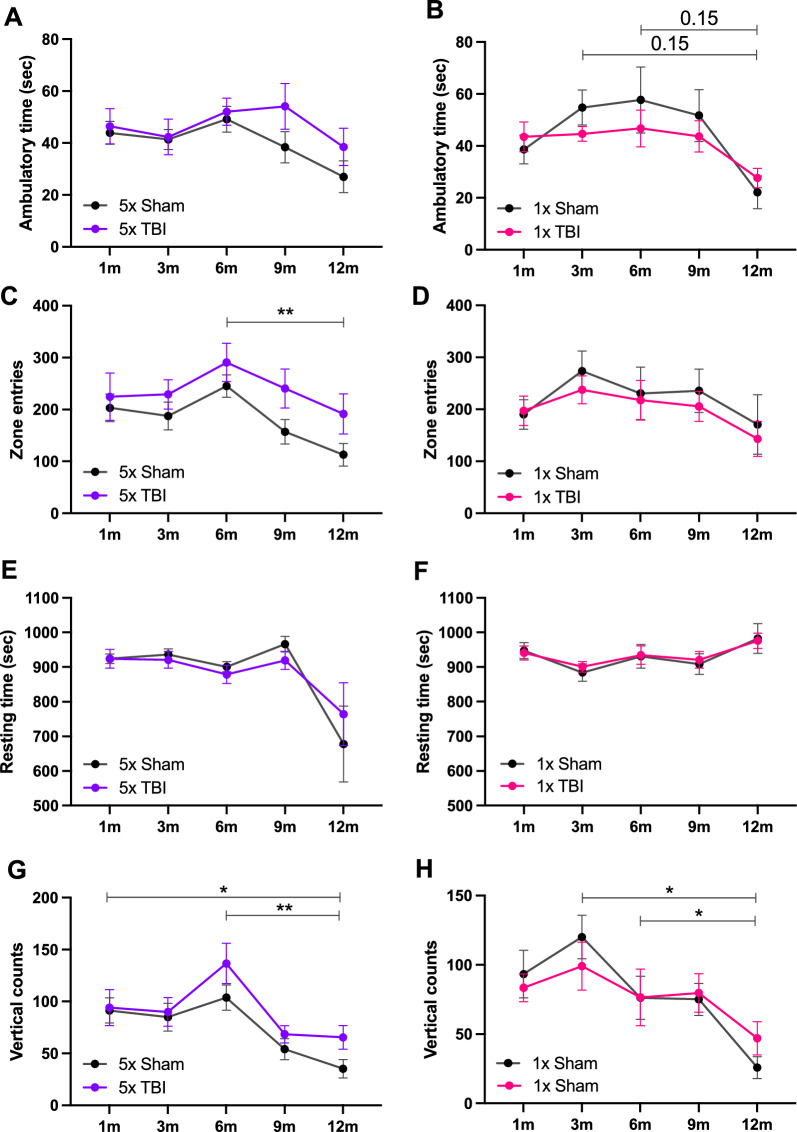
Locomotor activity in mice following single or r-mTBI. Locomotor activity following five (**A, C, E** and **G**) and one (**B, D, F** and **H**) impact at 1, 3, 6, 9 and 12 months post-injury . There were no significant changes in all four parameters between TBI and Sham animals across both injury groups. On average (not significant), **A** 5x TBI animals spent more time being ambulatory than Shams, **B** 1x TBI animals spent less time being ambulatory than Shams, **C** 5x TBI animals made more zone entries than Shams, **D** 1x TBI animals made less zone entries than Shams, **E** and **F** no major differences in resting time for 5x and 1x TBI animals compared to their respective Shams, **G** 5x TBI animals made more vertical counts than Shams, **(H)** 1x TBI animals on average made less vertical counts up to 6 months which was reversed up to 12 months post-injury. Mixed effects analysis with Holm-Sidak post-hoc for all graphs except for (**D** and **F**) which were analysed by two-way repeated measures ANOVA due to no missing values. Data expressed as mean ± SEM, *P < 0.05, **P < 0.01, n = 8–10/group, * indicates a significant difference within Sham animals across time points

For zone entries following five impacts, there was a significant effect of time post-injury (*F*_3.375,59.90_ = 3.590, *P* = 0.0152) and treatment group (*F*_1,18_ = 5.982, *P* = 0.0250), but no interaction effect (Fig. 9C). Post hoc analysis revealed no differences between 5x TBI animals and Shams, however there was a significant decrease in zone entries in the Sham group from 6 to 12 months post-injury (*t*_9_ = 5.554, *P* = 0.0035). Following one impact, there was no effect of time post-injury, treatment group or interaction between groups (Fig. 9D). It was interesting to note that 5x TBI animals made on average more zone entries than Shams (not significant), whereas 1x TBI animals made on average less zone entries compared to their Sham counterparts (not significant).

For resting time following five impacts, there was a significant effect of time post-injury (*F*_1.351,30.05_ = 7.091, *P* = 0.0072) but no treatment group or interaction effect. Post hoc analysis revealed no significant differences between groups across all time points (Fig. 9E). Following one impact, there was no effect of treatment group, time post-injury, subject or interaction (Fig. 9F).

Lastly, for vertical counts following five impacts, there was a significant effect of time post-injury (*F*_2.961,51.07_ = 8.957, *P* < 0.0001), but no treatment group or interaction effect. Post hoc analysis revealed no differences between treatment groups across all time points. However, there was a significant decrease in vertical counts within Shams from 1 to 12 (*t*_9_ = 3.615, *P* = 0.0494) and 6–12 (*t*_9_ = 5.100, *P* = 0.0064) months post-injury (Fig. 9G). Following one impact, there was a significant effect of time post-injury (*F*_3.200,68.81_ = 6.202, *P* = 0.0007) but no treatment group or interaction effect. Post hoc analysis revealed no differences between treatment groups across all time points, however there was a significant decrease in vertical counts within Shams from 3 to 12 (*t*_7_ = 4.296, *P* = 0.0318) and 6–12 (*t*_6_ = 4.781, *P* = 0.0302) months post-injury (Fig. 9H). Again, we note a reversed trend between 5x and 1x injury groups, albeit to a lesser extent for this parameter, with 5x TBI animals making on average more vertical counts than Shams (not significant). Taken together, this data suggests that five impacts causes on average an increase in ambulatory time, zone entries and vertical counts and a decrease in resting time, with the opposite trend occurring for the 1x TBI animals, but these data did not reach statistical significance. The finding that this pattern is reversed depending on impact number is interesting and may suggest the emergence of a hyperactive phenotype in the 5x TBI animals.

## Gait deficits are exacerbated with an increasing number of impacts

**Fig. 10:**
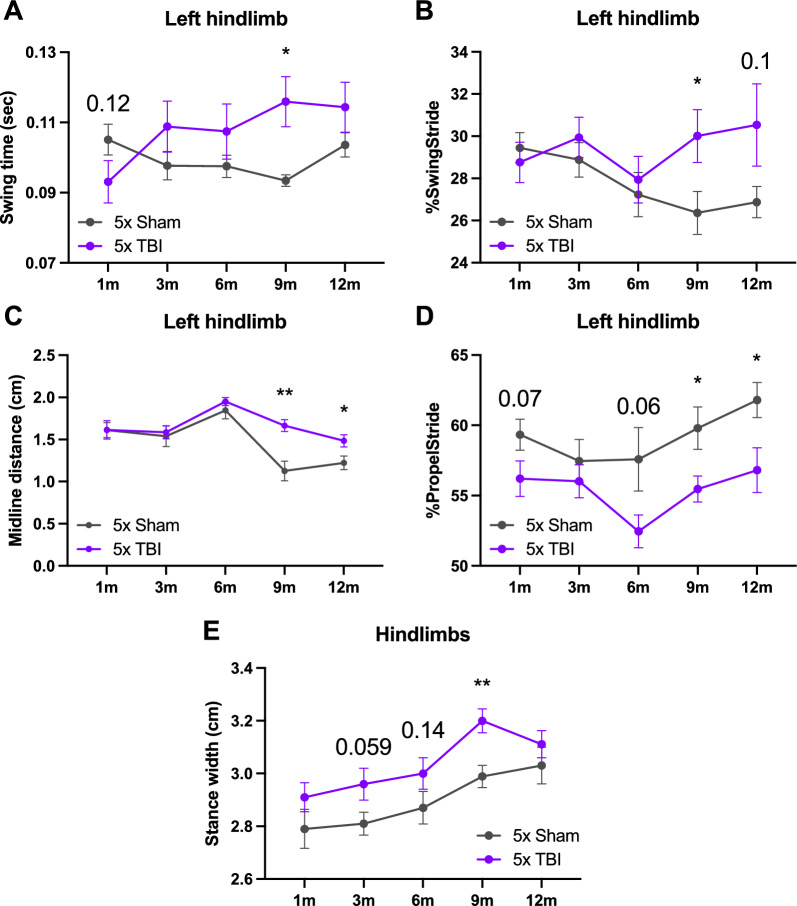
Assessment of gait in mice following single or r-mTBI. Gait deficits assessed using DigiGait following five impacts at 1, 3, 6, 9 and 12 months post-injury. **A** Swing is significantly increased at 9 months following 5x TBI compared to Shams. **B** %SwingStride is significantly increased at 9 months following 5x TBI compared to Shams. **C** Significant increase in midline distance at 9 and 12 months following 5x TBI versus Shams. **D** %PropelStride is significantly decreased at 9 and 12 months following 5x TBI versus Shams. **E** Significant increase in stance width at 9 months following 5x TBI compared to Shams. Mixed-effects analysis and uncorrected Fisher’s LSD post-hoc. Data expressed as mean ± SEM, *P < 0.05, **P < 0.01, n = 9–10 (Sham), n = 8–10 (TBI), * indicates a significant difference between TBI and Sham animals at a specific time point

Gait function was assessed using the DigiGait apparatus at 1, 3, 6, 9 and 12 months post-injury. For swing time in the left hindlimb following five impacts, there was a significant effect of treatment group (*F*_1,18_ = 5.568, *P* = 0.0298) and interaction of treatment group x time post-injury (*F*_4,70_ = 2.516, *P* = 0.0490) but no time post-injury effect. Post hoc analysis revealed a trend towards decreased swing time in 5x TBI animals compared to Shams initially at 1 month post-injury (*t*_16.38_ = 1.612, *P* = 0.1260) (Fig. 10A). However, swing time increased progressively thereafter in 5x TBI animals but remained relatively constant for Shams. Importantly, there was a significant increase in swing time at 9 months post-injury (*t*_9.914_ = 3.078, *P* = 0.0118) in 5x TBI animals versus Shams, demonstrating that repeated impacts cause the animals to swing their left hindlimb more at the chronic phase post-injury (Fig. 10A).

We investigated whether there were differences in %SwingStride, which is the percentage of a total stride duration that the paw is in a swing phase (in the air). In the left hindlimb following five impacts, there was a significant effect of treatment group (*F*_1,89_ = 5.967, *P* = 0.0166), but no time post-injury or interaction effect (Fig. 10B). Post hoc analysis revealed a significant increase in %SwingStride at 9 months post-injury (*t*_17.31_ = 2.258, *P* = 0.0372) and a trend towards increased %SwingStride at 12 months post-injury (*t*_10.31_ = 1.757, *P* = 0.1085) in 5x TBI animals versus Shams. Interestingly, while %SwingStride progressively decreased over time in Shams, it progressively increased in 5x TBI animals. This parameter typically increases with increasing speed, therefore this may suggest an increase in speed following five impacts perhaps due to the hyperactivity observed in this group on the locomotor test.

For midline distance, which refers to the distance between the centre of a paw to the midline of the animals’ body, in the left hindlimb following five impacts there was a significant effect of time post-injury (*F*_3.245,70.57_ = 11.26, *P* < 0.0001), treatment group (*F*_1,87_ = 10.81, *P* = 0.0015) and a significant interaction of time post-injury x treatment group (*F*_4,87_ = 2.814, *P* = 0.0301) in the left hindlimb following five impacts (Fig. 10C). Post hoc analysis revealed no significant differences in midline distance from 1 to 6 months post-injury between treatment groups, however as time progressed, 5x Sham mice shortened their midline distance (perhaps an effect of age or learning) although this was not the case for 5x TBI mice. Hence, midline distance was significantly higher at 9 (*t*_14.39_ = 3.965, *P* = 0.0013) and 12 (*t*_16.91_ = 2.435, *P* = 0.0263) months post-injury in 5x TBI animals versus Shams. This indicates that five repeated impacts causes an increase in left hindlimb extension which may suggest weakness in that limb and importantly that this deficit develops at the chronic stage following r-mTBI (Fig. 10C).

We also noted differences in the %PropelStride parameter, which refers to the percentage of a stride phase in which the paw is in a propulsion phase (paw off the ground). In the left hindlimb following five impacts, there was a significant effect of treatment group (*F*_1,89_ = 17.86, *P* < 0.0001) but no interaction or time post-injury effect. Post hoc analysis revealed strong trends towards decreased %PropelStride at 1 (*t*_17.65_ = 1.865, *P* = 0.0788) and 6 (*t*_13.43_ = 2.013, *P* = 0.0646) months, and a significant decrease at 9 (*t*_14.95_ = 2.450, *P* = 0.0271) and 12 (*t*_15.60_ = 2.465, *P* = 0.0257) months post-injury in 5x TBI animals versus Shams (Fig. 10D). This demonstrates that r-mTBI causes a reduction in paw propulsion during a stride, further suggesting weakness of the left hindlimb at the chronic stage following r-mTBI.

For stance width of both hindlimbs following five impacts there was a significant effect of time post-injury (*F*_3.739,65.43_ = 9.079, *P* < 0.0001) and treatment group (*F*_1,18_ = 8.373, *P* = 0.0097) but no interaction effect (Fig. 10E). Post hoc analysis revealed a strong trend towards increased stance width at 3 months (*t*_16.38_ = 2.027, *P* = 0.0593), a small trend at 6 months (*t*_*17.98*_ = 1.517, *P* = 0.1467) and a significant increase at 9 months (*t*_17_ = 3.429, *P* = 0.0032) post-injury in 5x TBI animals versus Shams. This indicates that repeated impacts causes an increase in stance width in the hindlimbs chronically post-injury, suggesting that these animals need to adjust their posture in order to increase their stability (Fig. 10E). In contrast, there were no differences observed for any of the parameters investigated following a single injury (data not shown), demonstrating that chronic gait impairments occur as a result of repeated but not a single injury.

### Mild anhedonia but no anxiety-like behaviour chronically post r-mTBI

**Fig. 11:**
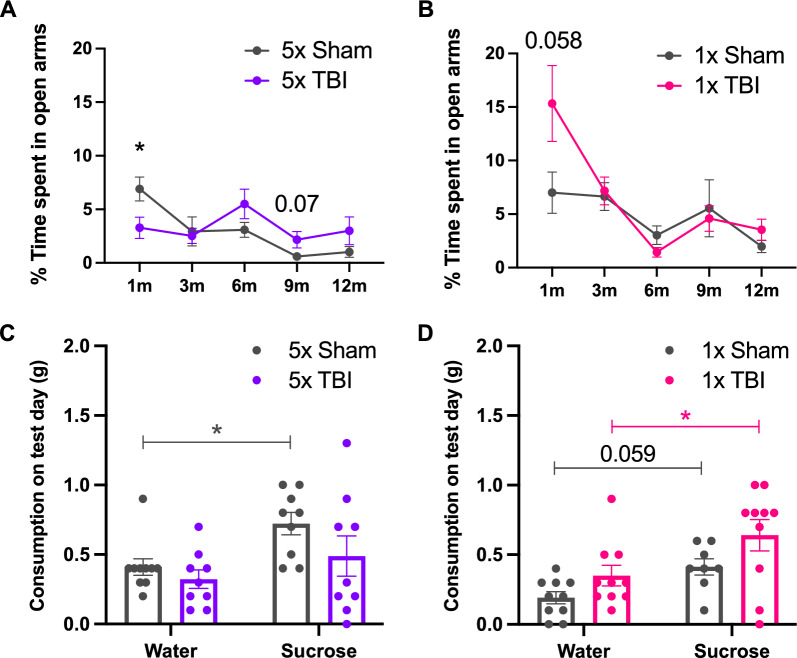
Anxiety-like and depressive-like behaviours in mice following single or r-mTBI. Assessment of anxiety-like behaviour on the Elevated Plus Maze (**A** and **B**) and depressive-like behaviour with the Sucrose Preference Test (**C** and **D**) following five (**A** and **C**) and one (**B** and **D**) impact at 1, 3, 6, 9 and 12 months post-injury (for the EPM) and 12 months post-injury only (for the SPT). Note that the 1-month time point presented in this figure (**A** and **B**) has been previously published elsewhere [44] and that the authors have obtained permission to re-use this data. **A** Significant decrease in time spent in the open arms following 5x TBI at 1 month post-injury. No significant changes thereafter. **B** Strong trend towards increased time spent in the open arms of the maze in the 1x TBI group compared to Shams at 1-month post-injury with no changes thereafter. **C** Significant increase in sucrose consumption in the Sham group only. **D** Significant increase in sucrose consumption in the 1x TBI group with a strong trend towards increased sucrose consumption in the Sham group. **A** and **B** Mixed-effects analysis with uncorrected Fisher’s LSD post-hoc. **C** and **D** Ordinary Two-way ANOVA with Holm-Sidak post-hoc. Data expressed as mean ± SEM, *P < 0.05, n = 9–10/group

Anxiety-like behaviour was assessed using the EPM at 1, 3, 6, 9 and 12 months post-injury and depressive-like behaviour, specifically anhedonia, was assessed using the SPT at 12 months post-injury only (Fig. 3). Focussing on the EPM following five impacts, there was a significant effect of time post-injury (*F*_3.040,48.64_ = 6.541, *P* = 0.0008), a significant interaction effect of treatment group x time post-injury (*F*_4,64_ = 3.955, *P* = 0.0063) but no effect of treatment group (Fig. 11A). Post hoc analysis revealed a significant decrease in time spent in the open arms of the EPM in the 5x TBI group as compared to Shams (*t*_15.81_ = 2.438, *P* = 0.0270) at 1-month post-injury, which is indicative of anxiety-like behaviour. Time spent in the open arms of the maze decreased over time in the Sham group. In the 5x TBI group, time spent in the open arms was elevated as compared to Shams from 6 to 12 months post-injury. In fact, there was a trend towards increased time spent in the open arms in the 5x TBI group as compared to Shams at 9 months post-injury (*t*_10.83_ = 1.958, *P* = 0.0765, Fig. 11A). This is interesting as it not only indicates an increase in willingness to explore but may also suggest an increase in risk-taking behaviour in the 5x TBI group, although further testing would be required to confirm this. Following one impact, there was a significant effect of time post-injury (*F*_2.056,35.46_ = 10.67, *P* = 0.0002) and a significant interaction of treatment group x time post-injury (*F*_4,69_ = 3.198, *P* = 0.0181) but no effect of treatment group. Post hoc analysis revealed a strong trend towards increased time spent in the open arms of the maze in the 1x TBI group compared to Shams at 1-month post-injury (*t*_13.74_ = 2.063, *P* = 0.0585, Fig. 11B) and there were no changes thereafter.

For the SPT at 12 months following five impacts, there was a significant effect of bottle choice on the test day (*F*_1,33_ = 6.746, *P* = 0.0139), but no treatment group or interaction effect. Post hoc analysis showed a significant increase in sucrose consumption as compared to drinking water for the Sham group (*t*_33.00_ = 2.426, *P* = 0.0414, Fig. 11C) but not for the 5x TBI group. At 12 months following one impact, there was a significant effect of bottle choice (*F*_1,34_ = 10.68, *P* = 0.0025) and treatment group (*F*_1,34_ = 6.108, *P* = 0.0186) on the test day but no interaction effect (Fig. 11D). Post hoc analysis revealed a significant increase in sucrose consumption as compared to drinking water for the 1x TBI group (*t*_34.00_ = 2.696, *P* = 0.0215) and a strong trend towards increased sucrose consumption as compared to drinking water in the Sham group (*t*_34.00_ = 1.950, *P* = 0.0594, Fig. 11D). These data indicate that both the 1x TBI group and their Sham counterparts had a preference for the sucrose water and as such do not display any evidence of anhedonia. On the other hand, whereas the Sham animals in the 5x injury group displayed a strong preference for the sucrose water, this preference was absent in the 5x TBI group, which suggests the presence of mild anhedonia as these mice do not show any clear preference between drinking water alone versus the sucrose water.

### Mild deficits in spatial learning and memory but not short-term memory at 12 months following five impacts

**Fig. 12:**
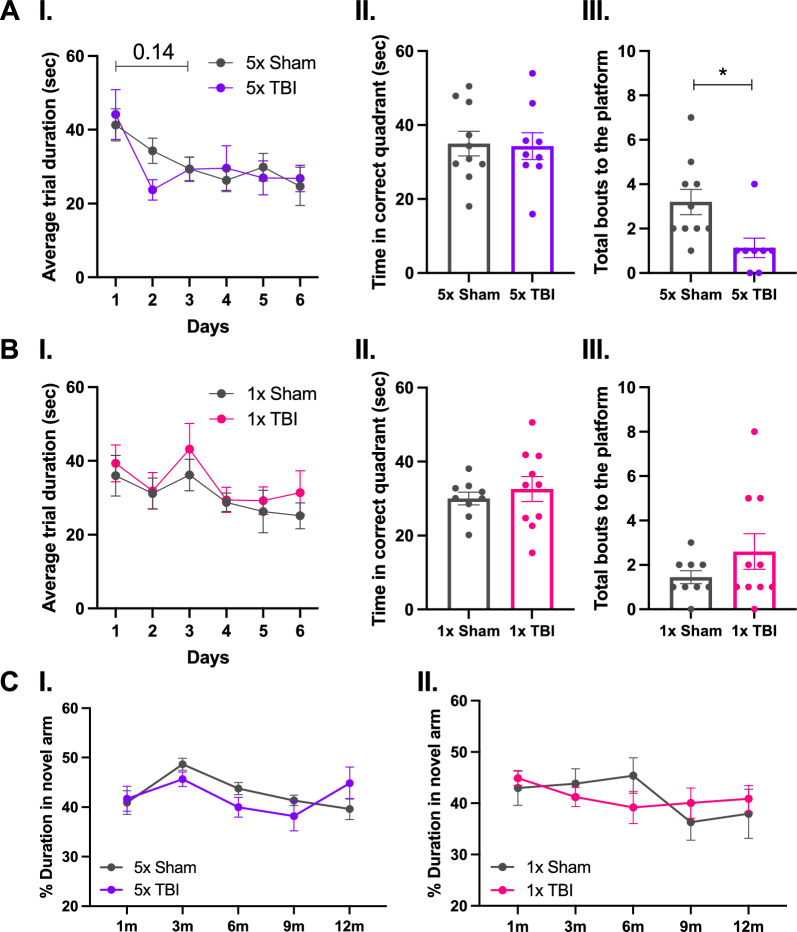
Cognitive testing in mice following single or r-mTBI. Assessment of spatial learning and memory on the Morris Water Maze (**A** and **B**) and short-term memory on the Y-maze (**C**) following five (**A** and **C-I**) and one (**B** and **C-II**) impact at 12 months (**A** and **B**) and 1, 3, 6, 9 and 12 months (**C**) post-injury. At 12 months following five impacts, there were (**A-I**) no significant changes in average trial duration across all days, (**A-II**) no differences in time spent in the correct quadrant on the Probe day but (**A-III**) there was a significant decrease in total bouts made to the platform on the Probe day in the 5x TBI group. At 12 months following one impact, there were (**B-I**) no changes in average trial duration across all days, (**B-II**) no differences in time spent in the correct quadrant on the Probe day and (**B-III**) no differences in total bouts made to the platform on the Probe day. No significant changes in time spent in the novel arm following (**C-I**) five impacts or (**C-II**) one impact across all time points post-injury. **A-I** Two-way repeated measures ANOVA with Hold-Sidak post-hoc, **B-I** mixed-effects analysis with Holm-Sidak post-hoc, (**A-II, III** and **B-II, III**) unpaired two-tailed Student’s t-test and (**C**) mixed-effects analysis with Holm-Sidak post-hoc. Data expressed as mean ± SEM, *P < 0.05, n = 7–10 (Sham), n = 8–10 (TBI)

We assessed whether five or one impact would result in spatial learning and memory deficits on the MWM at 12 months following the last impact. At 12 months following five impacts, there was a significant effect of day (*F*_3.534,60.07_ = 4.286, *P* = 0.0056), but no effect of treatment group, subject or interaction effect (Fig. 12A-I). Post hoc analysis revealed no significant differences in average trial duration across all days of the trial between treatment groups, although there was a small trend towards decreased average trial duration in the 5x Sham group from day 1 versus 3 (*t*_9_ = 3.247, *P* = 0.1406, Fig. 12A-I). There were no significant differences in time spent in the correct quadrant (where the platform had been located) on the Probe day (Fig. 12A-II). However, there was a significant decrease in total bouts made to the platform region on the Probe day in the 5x TBI group compared to Shams (*t*_16_ = 2.750, *P* = 0.0142, Fig. 12A-III). This demonstrates that although the 5x TBI animals remembered which quadrant the platform was located in, they were not able to locate it, suggesting a mild deficit in spatial learning and memory. In fact, 5x TBI (but not 1x TBI) mice displayed a mild trend towards increased swim speeds the day prior to the probe trial (*t*_17_ = 1.679, *P* = 0.1114, Additional file 1: Fig. SA). Conversely, at 12 months following one impact, there was a significant effect of day (*F*_3.899,67.06_ = 2.669, *P* = 0.0408) but no treatment group or interaction effect. Post hoc analysis showed no significant differences in average trial duration across all days of the test (Fig. 12B-I). Similarly, there were no differences in time spent in the correct quadrant (Fig. 12B-II) or total bouts made to the platform region (Fig. 12B-III) on the Probe day. Taken together, these data demonstrate that spatial learning and memory deficits, as assessed by the MWM, develop mildly at 12-months following five impacts only. Furthermore, the Y-maze was used to assess short-term memory function at 1, 3, 6, 9 and 12 months post-injury. Following five impacts, there was a significant effect of time post-injury (*F*_3.643,61.02_ = 3.810, *P* = 0.0097) but no treatment group or interaction effect. Following one impact, there was no treatment group, time post-injury or interaction effect. Post hoc analysis revealed no significant differences in time spent in the novel arm across all time points and in both injury groups (Fig. 12C-I and II). These data demonstrate that, as assessed by the Y-maze, neither five nor a single injury resulted in short-term memory deficits.

## Discussion

The primary goal of this study was to determine whether a single or r-mTBI leads to changes in metal levels and proteins consistent with the onset/progression of neurodegeneration at 12-months post-injury, long-term behavioural deficits and additionally whether r-mTBI leads to changes in gene expression at 6-months post-injury.

### No changes in gene expression following r-mTBI

Gene expression studies at 6-months following r-mTBI are relatively scarce. We found no DEGs as a function of r-mTBI, and our data revealed sex as the greatest source of variation. This lack of transcriptional changes at 6-months post-injury was surprising since we observed marked translational changes at 12-months post-injury. Indeed, it is possible that gene expression changes between treatment groups were masked by the sex variable noise. Indeed, the stripchart of the most DEG (Tbc1d19) between treatments groups suggested that separation of sexes may reveal differences between treatment groups. The Tbc1d19 gene is predicted to act as a GTPase-activating protein (GAP) for Rab proteins and is relatively uncharacterised in neurodegenerative diseases or TBI [58]. One AD study performed RNA interference screening of human GTPases in HeLa cells expressing the Swedish APP mutation and found that multiple RabGAP proteins (although not Tbc1d19) decreased APP and Aβ levels, suggesting that these proteins affect Aβ production [59].

It is possible that gene expression changes were localised to specific cell types which our bulk RNA sequencing analysis would have missed, and more cell-specific genetic analyses such as single-cell RNA sequencing could be informative. The 6-month timepoint may also have been too late to detect significant changes since several single mTBI and r-mTBI studies have reported DEGs at acute and sub-acute timepoints post-injury [60]. Therefore, we acknowledge that future experiments may need to focus on earlier timepoints.

### Alterations in brain iron content and iron-regulatory proteins following r-mTBI

The primary pathogenic contributor that we interrogated in this study is metal ion dyshomeostasis. Indeed, changes in brain iron metabolism can be due to a dysregulation in the expression of iron-regulatory proteins [7]. In the ipsilateral hemisphere, we observed a significant decrease in TfR levels and an increase in ferritin levels following five impacts, likely demonstrating the iron-loading capacity of ferritin in decreasing the labile iron pool. This finding is important as ferritin is closely related to AD pathology, in fact a recent study found that increased ferritin in the cerebrospinal fluid (CSF) of AD patients with mild cognitive impairment was a predictor of long-term cognitive decline [61]. Collectively, r-mTBI caused a reduction in TfR, an increase in DMT1 and ferritin, and a non-significant reduction in ferroportin in the ipsilateral hemisphere at 12-months post-injury, which may suggest reduced iron transport into the cell, increased cytosolic iron import and increased intracellular iron storage, respectively. Importantly, the observed ipsilateral changes in iron-regulatory proteins are molecular signatures indicating excessive brain iron levels, which was interesting given the lack of changes in brain iron content within this hemisphere. Therefore, this suggests that iron levels may have been increased in the ipsilateral hemisphere prior to the 12-month timepoint. Although our study did not capture this hypothesised change in ipsilateral iron content, this data likely demonstrates the ability of this hemisphere to respond to changes in iron content and adequately restore iron metabolism.

Interestingly, while no changes in iron-regulatory proteins were reported contralaterally, a prominent increase in brain iron content was seen, suggesting two possibilities. First, that these iron-regulatory proteins may be altered shortly after the 12-month timepoint in response to excessive iron levels or second, that the lack of iron-regulatory protein changes may reflect a failure of the regulatory machinery in responding to excessive iron levels and restoring homeostasis. This is interesting as we previously reported a significant increase in contralateral iron levels as measured by laser ablation-ICP-MS (LA-ICP-MS) in the cortex of young-adult (4-month-old) mice at 1-month following five impacts [8]. Although we have not performed a longitudinal characterisation of iron levels between the 1- and 12-month timepoints, these data indicate that r-mTBI causes an early contralateral increase in brain iron levels which persists up to 12-months post-injury, indicating widespread brain pathology in this r-mTBI model. Indeed, these changes in iron-regulatory proteins are consistent with what is seen in several neurodegenerative diseases where an increase in iron is a prominent feature [30, 62]. However, while it is possible that a change in iron levels causes a change in iron-regulatory proteins (as proposed here), we also note the possibility that a change in iron-regulatory proteins may cause a change in iron levels, and the sequence of these events are actively being debated in the field. It is also possible that certain cell types (e.g. glia) may be sequestering more iron, and that the associated iron pathology may not be reflected in the total iron assessed and would need to be more specifically studied. Indeed, an earlier study reported an increase in iron-positive cells, assessed via Perl’s reaction for iron staining, in the brain of human TBI patients 3–17 h post-trauma and the authors report that the majority of the iron-positive cells were in glia-like cells [63]. Although the focus of this study was iron and its regulatory proteins, we also report the changes observed in zinc and copper as these metals are also involved in the pathogenesis of several neurodegenerative diseases and have also been previously found to be altered acutely in a single TBI model [42]. These data are interesting given that zinc overload can contribute to the cognitive deficits observed in AD [64] and PD [65]. Moreover, there is also accumulating data highlighting the relationship between neuronal injury and excess zinc post-TBI [66]. For example, a previous study showed that zinc was released from pre- to postsynaptic neurons following a single TBI which contributed to neuronal death [66]. Importantly, this effect was mitigated following treatment with zinc chelators. Although neuronal injury was not examined in the current study, it is a hallmark pathology of TBI and we previously reported significant neuronal loss at the injury site at 1-month following r-mTBI [44]. Furthermore, we note the possibility that the significantly increased zinc observed herein may have contributed, in part, to the mild cognitive deficits observed in this study. In addition, copper is heavily involved in the formation of ROS in AD [67] and can also contribute to neuronal death [68]. Copper has also been shown to contribute to depressive-like behaviours in both human patients [69] and animal models [70], as well as contributing to movement disorders [71]. Given that we observed mild anhedonia and significant gait deficits chronically post-injury, then it is possible that copper may have, at least in part, contributed to these behavioural changes. Future experiments would benefit from characterising the expression of zinc and copper-regulatory proteins in the context of single and r-mTBI.

These findings have significant implications for the r-mTBI field. Firstly, iron accumulation following r-mTBI has, to the best of our knowledge, only been reported in a handful of studies [72–74]. However in most of these studies, the authors’ primary research aims did not involve directly assessing brain iron level changes as a potential driver of neurodegeneration following r-mTBI (nor were they discussed in this way), rather it seems that iron deposition was used as an indicator of small blood vessel leakage due to injury. In a more recent study, Wang and colleagues subjected mice to r-mTBI (4 hits every 48 h) and report a significant increase in brain iron levels at 7, 14, 28 and 42 days post-injury relative to shams, with iron levels peaking on day 28 post-injury [75]. The authors also report a significant decrease in ferroportin at 1, 3, 7, 14, 28 and 42 days post-injury and a significant increase in TfR 28 days post-injury compared to shams [75]. Although this study was conducted at a different timepoint than that presented herein, this is interesting as it suggests a reduction in iron export and an increase in iron transport into the cell at 28 days post-injury likely leading to the increase in brain iron levels. Therefore, the results from this study in combination with our previous work [8] provide the first evidence demonstrating a sub-acute (1-month) increase in brain iron levels which extends to the chronic (12-month) stage in the contralateral hemisphere, demonstrating that r-mTBI causes widespread iron pathology. Secondly, our finding of brain iron accumulation is of particular significance as it can contribute to toxicity by promoting oxidative stress, neuronal death, inflammation, mitochondrial dysfunction, protein aggregation and excitotoxicity [7, 30, 76, 77] and importantly, iron accumulation contributes to cognitive impairment [78]. Therefore, it is possible that the cognitive deficits observed in this study may in part be due to iron overload, suggesting that therapeutic strategies aimed at restoring homeostatic iron levels may be beneficial. In fact, administration of the iron chelator deferoxamine, which binds and removes excess iron, reduced the TBI-induced increase in iron levels and iron-storing proteins, brain atrophy and improved spatial learning and memory at 28 days following a single TBI in rats [79]. Moreover in a recent study, the HBED iron chelator [80] significantly reduced motor impairments, cortical injury volume, hippocampal swelling, microgliosis and oxidative stress markers in a mouse model of single CCI. Lastly, iron chelation has also demonstrated beneficial effects in several neurodegenerative disorders such as AD [81], PD [82] and amyotrophic lateral sclerosis (ALS) [83]. In fact, we have recently shown that the iron chelator deferiprone improves neuropathology and behavioural outcomes following a single TBI [84]. Collectively, the evidence suggests that iron chelation may be beneficial in reducing numerous pathological outcomes of TBI, many of which are known to promote neurodegeneration. As iron chelation has yet to be trialled following r-mTBI, our study highlights the need to test metal-targeting compounds in animal models of r-mTBI.

## Tau protein following single and r-mTBI

Our results demonstrate that evidence for tau pathology, either through a direct increase in tau phosphorylation or alterations in tau-regulatory proteins, was not present at 12 months post-injury in this r-mTBI model. There was however a significant contralateral increase in T22 oligomeric tau at 12 months following a single injury, which was curious given that we observed no changes in tau following r-mTBI. This was interesting given the presence of cognitive/motor deficits in this group at 12 months post-injury and since we previously reported trends towards increased tau phosphorylation and significant changes in tau-regulatory proteins at 1-month following r-mTBI [44].

It is important to note that in contrast to the data presented herein, a number of studies in the literature have reported significant changes in tau phosphorylation following r-mTBI at acute and sub-acute timepoints, although significantly fewer studies report tau phosphorylation changes at chronic time points and particularly at 12-months post-injury. For instance, in a study by Ojo and colleagues where 18-month-old mice expressing human tau (hTau) received a single or five r-mTBIs (delivered 48 h apart), the authors reported a significant increase in tau phosphorylation in the motor/somatosensory cortex and hippocampus at 3 weeks following r-mTBI compared to a single TBI, demonstrating that changes in tau pathology were present up to 3 weeks post-injury [26]. A recent 2021 study used the CCI model to subject 8–10 week old wild-type mice to five r-mTBIs (one/day for 5 days) [85]. While there was no tau pathology detected at 1-week post-injury, significant AT8 immunoreactivity was found in the cortex and hippocampus at 4 and 10 weeks post-injury in the r-mTBI group as compared to the uninjured control group. In comparison to the previous study, then this study suggests that wildtype mice may require a longer timeframe for tau pathology to develop. Moreover in a longer term study, Petraglia and colleagues used a modified CCI model where mice received either a sham procedure, a single injury or 6 hits per day over a period of 7 days (receiving a total of 42 hits/animal) [27]. At 7 days and 1-month post-injury, the authors found a significant increase in tau phosphorylation in both single and r-mTBI groups. Importantly, tau phosphorylation persisted up to 6 months post-injury in the r-mTBI group only (and not in the single TBI or sham groups), suggesting that repetitive injuries may trigger pathological tau species to persist for months following the last impact. However, it is important to note that there are several differences between the previous study and the study presented herein which may likely explain the differing results. Indeed, Petraglia and colleagues injured the mice more frequently (several times a day and for several days) as compared to our five-hit paradigm (one injury every 48 h for a total of five impacts). It is possible that injuring the brain several times a day, before it has been able to recover, may exacerbate the resulting pathology. In fact, injury frequency has been previously shown to affect cerebral vulnerability and thus the outcomes post-TBI. For instance, one study investigated the cerebral metabolic rate of glucose (CMRglc, which is typically decreased following injury) in rats following sham, single TBI and two mTBIs at 24 and 120 h intervals [86]. The authors showed that a single TBI caused a 19% hippocampal and cortical reduction in CMRglc at 24 h post-injury, which was further decreased by 36% 24 h later when a second hit was delivered and which remained decreased by 25% up to 3 days post-injury. Conversely, when the second hit was delivered after CMRglc levels had normalised, then the reduction in CMRglc remained comparable to that following a single TBI. As such, this study provides evidence that injury frequency can impact upon the resulting pathological outcomes in r-mTBI studies. Therefore it is possible that our injury paradigm may not have been frequent enough for the animals to develop significant tau pathology.

Taking these studies into consideration, then the lack of significant tau phosphorylation changes at 12-months post-injury reported in this study is noteworthy. Importantly, our results are in line with previous and more recent research from the authors who developed this r-mTBI model [28, 29], where chronic neurobehavioural impairments are present without tau pathology. For instance, Mouzon, et al. [28] reported significant spatial learning and memory deficits using the Barnes maze at 6- and 12-months following r-mTBI but not following a single injury and similarly there was a lack of tau pathology at both time points and injury groups. They later showed that r-mTBI in transgenic hTau mice led to learning and memory impairments that worsened from 2-weeks to 12-months post-injury but only transient changes in tau phosphorylation were observed 24 h post-injury, which did not extend to the chronic (12-month) stage post-injury [29]. Our replication of these results only further question whether tau is a critical player in the development of the chronic behavioural sequelae post-injury. However, we cannot exclude the possibility that significant changes in tau and tau-regulatory proteins may have occurred earlier on, such as at 3- or 6-months post-injury, which were not investigated for tau pathology in this study. Indeed, it is possible that an earlier increase in tau phosphorylation may have led to the development of chronic behavioural deficits.

## Chronic behavioural alterations following single and r-mTBI

Our results confirmed that this model of r-mTBI recapitulated several clinically relevant behavioural impairments characteristic of human r-mTBI, specifically the associated neurodegenerative condition CTE, such as motor impairments, slight hyperactivity, mild anhedonia and learning/memory deficits. The lack of neurological deficits chronically following r-mTBI, assessed via the mNSS test, suggests that neurological deficits occur early on following injury [44], plateauing shortly thereafter and up to 12-months post-injury.

The reversal of trends on the Locomotor test between both injury groups suggests a slight hyperactive phenotype following r-mTBI, which is commonly reported in TBI [87, 88]. These results in parallel with the observed gait deficits provide further insights. The left hindlimb was particularly affected following r-mTBI, which was expected as the injury was delivered onto the right parietal cortex. Given that %SwingStride typically increases with increasing speed (DigiGait Imaging System Indices, Mouse Specific, Inc), this suggests that five impacts increase walking speed (despite belt speed remaining constant), indicating hyperactivity [89]. The significant increase in midline distance and stance width indicates hyperextension of the left hindlimb at 9- and 12-months post-injury, highlighting the need to make postural adjustments to maintain stability. Overall, r-mTBI led to a chronic weakness in both hindlimbs (but particularly the left) post-injury, a common finding in human TBI [90]. Although we previously reported anxiety-like behaviour following r-mTBI sub-acutely post-injury [44], this did not extend to chronic timepoints. However, due to repeated EPM testing, we acknowledge the possibility of a learning effect or a loss of interest in the task. Interestingly, r-mTBI led to more time spent in the open arms from 6 to 12 months suggesting increased exploratory behaviour, which has been previously reported at 3-months following r-mTBI [91, 92]. However, this could also suggest an increase in risk-taking behaviour, with a recent study reporting increased risk-taking behaviour via increased duration in the open arms at 5-months following a single TBI [93]. This is important as executive dysfunction (specifically disinhibition) is a common consequence of CTE [94]. Additionally, r-mTBI mice experienced mild anhedonia at 12-months post-injury, which has previously been reported in models of single and r-mTBI [95, 96] and is a well-established consequence of CTE [94].

Despite no changes in short-term memory on the Y-maze, r-mTBI (but not a single injury) caused a mild spatial learning and memory deficit on the MWM. Indeed, both 5x TBI animals and Shams spent an equal amount of time in the correct quadrant, demonstrating their ability to engage in an efficient search strategy. However, 5x TBI mice made significantly less bouts to the platform suggesting that they were able to use the visual cues to recall the general vicinity of the platform, but were unable to determine its specific location in comparison to Shams. This was likely not due to the gait impairments observed in this group, as 5x TBI mice displayed a non-significant increase in swim speed on the day prior to the probe trial. These findings are consistent with several experimental r-mTBI studies reporting spatial learning and memory deficits at the chronic stage post-injury [91, 97, 98] and translates to human TBI cases, particularly CTE [99]. However, we note that all mice learned the task relatively quickly, therefore it is possible that reduced bouts to the platform may also be interpreted as a loss of interest in the task. To overcome this in the future, the authors suggest using variations of the MWM task such as reducing the number of testing days or using the reversal learning paradigm [100].

## Conclusion

In conclusion, we have conducted an extensive behavioural, biochemical and metallomic characterisation of a r-mTBI mouse model, where we found significant behavioural deficits up to 12 months post-injury, no evidence of tau pathology but significant evidence of dysregulated iron metabolism following r-mTBI. We also report a lack of changes in gene expression at 6-months following r-mTBI, and we suggest that further analysis of male and female mice separately may be valuable. To the best of our knowledge, we are the first to describe changes in iron and its regulatory proteins in a mouse model of r-mTBI at a chronic stage post-injury. This is an important finding as it provides further evidence for the implication of iron in the chronic sequalae of r-mTBI, an area of research that has been largely unexplored in the field. Importantly, this work highlights iron dyshomeostasis as a potential novel secondary injury mechanism contributing to the chronic pathophysiology of r-mTBI. Future studies should focus on investigating the status of iron metabolism within the brain following r-mTBI, possibly through the investigation of oxidative stress and lipid peroxidation markers which may provide clues into the involvement of ferroptosis (or other iron-mediated mechanisms) in the development of neurodegeneration following r-mTBI. Furthermore, this work highlights the potential of a new avenue of therapeutic targeting in the r-mTBI field, with metal chaperone and chelating drugs already in clinical trials for several neurodegenerative disorders such as AD [101] and PD [102] but which have yet to be trialled in single/r-mTBI or even CTE.

## Supplementary Information

**Additional file 1:** **Figure S.1.** Bulk metal analysis in mice receiving r-mTBI only. **Figure S.2.** Bulk metal analysis in mice receiving single mTBI only. **Figure S.3.** Swim speed of mice receiving a single or r-mTBI.

## Data Availability

All data generated or analysed in this study are included in this published article (and its supplementary files). The RNA sequencing dataset generated and analysed during the current study is available in the GitHub repository (https://github.com/sydneyjuan/RNAseq_r-mTBI/blob/main/r-mTBI_DGEanalysis_SJ.Rmd).

## Abbreviations

AD: Alzheimer’s disease
ALS: Amyotrophic lateral sclerosis
APP: Amyloid precursor protein
ARC: Animal resources centre
CCI: Controlled cortical impact
CDK5: Cycline-dependent kinase 5
CMRglc: Cerebral metabolic rate of glucose
CPM: Counts per million
CSF: Cerebrospinal fluid
CTE: Chronic traumatic encephalopathy
DEG: Differentially expressed genes
DGE: Differential gene expression
DMT1: Divalent metal transporter-1
ELISA: Enzyme-linked immunosorbent assay
EPM: Elevated plus maze
ERK1/2: Extracellular signal-regulated kinases 1/2
FDR: False discovery rate
GAP: GTPase-activating protein
GSK3β: Glycogen synthase kinase 3β
HPC: High performance computing
hTau: Human tau
ICP-MS: Inductively coupled plasma-mass spectrometry
LA-ICP-MS: Laser ablation-inductively coupled plasma-mass spectrometry
MDS: Multidimensional scaling
mNSS: Modified neurological severity score
mTBI: Mild traumatic brain injury
MWM: Morris water maze
PBS: Phosphate buffered saline
PD: Parkinson’s disease
PKC: Protein kinase C
PP1: Protein phosphatase 1
PP2A: Protein phosphatase 2 A
PP2B: Protein phosphatase 2B
QC: Quality control
r-mTBI: Repetitive mild traumatic brain injury
RQS: RNA quality score
RT-qPCR: Reverse transcription polymerase chain reaction
SPT: Sucrose preference test
TBI: Traumatic brain injury
TBST: Tris-buffered saline
Tf: Transferrin
TfR: Transferrin receptor
