# Supplementary material for: Alterations in iron content, iron-regulatory proteins and behaviour without tau pathology at one year following repetitive mild traumatic brain injury
Source: Acta Neuropathol Commun. 2023 Jul 18;11:118. doi: 10.1186/s40478-023-01603-z (PMC10353227; doi:10.1186/s40478-023-01603-z)
Supplement: Supplementary file 1 — Additional file 1: Figure S.1. Bulk metal analysis in mice receiving r-mTBI only. Figure S.2. Bulk metal analysis in mice receiving single mTBI only. Figure S.3. Swim speed of mice receiving a single or r-mTBI. [file 40478_2023_1603_MOESM1_ESM.docx]

**Supplementary data**

**Figure S.1.** Bulk metal analysis in mice receiving r-mTBI only. ICP-MS analysis of zinc **(A and C)** and copper **(B and D)**

in the ipsilateral **(A and B)** and contralateral **(C and D)** hemispheres of the parietal cortex (injury site) at 12 months following five impacts. No significant changes in zinc **(A)** or copper **(B)** levels in the ipsilateral hemisphere following five impacts. Significant increase in zinc **(C)** and copper **(D)** levels in the contralateral hemisphere following five impacts. Unpaired two-tailed Student’s t-test. Data expressed as mean ± SEM, ****P* < 0.001, n= 9 (Sham), n= 8-9 (TBI).

**Figure S.2.** Bulk metal analysis in mice receiving single mTBI only. ICP-MS analysis of iron **(A and D)**, zinc **(B and E)** and copper

**(C and F)** in the ipsilateral **(A, B and C)** and contralateral **(D, E and F)** hemispheres of the parietal cortex (injury site) at 12 months following one impact. There were no changes in iron **(A)**, but there was a significant decrease in zinc **(B)** and a significant increase in copper **(C)** levels in the ipsilateral hemisphere following one impact. No significant changes in iron **(D)**, zinc **(E)** or copper **(F)** levels in the contralateral hemisphere following one impact. Unpaired two-tailed Student’s t-test. **P* < 0.05, ***P* <0.01, data expressed as mean ± SEM, n= 10 (Sham), n= 9-10 (TBI).

**Figure S.3.** Swim speed of mice receiving a single or r-mTBI. Velocity (mm^s^) of the mice following five impacts **(A)** and a single impact

**(B)** on day 6 of the MWM, one day prior to the Probe day, at 12-months post-injury. **(A)** Mild trend towards increased swim speed following 5x TBI. **(B)** No significant changes in swim speed following 1x TBI. Unpaired two-tailed Student’s t-test. Data expressed as mean ± SEM, **P* < 0.05, n= 10 (Sham), n= 9-10 (TBI).
